# Systemic prime exacerbates the ocular immune response to heat-killed Mycobacterium tuberculosis

**DOI:** 10.1016/j.exer.2022.109198

**Published:** 2022-07-31

**Authors:** Kathryn L. Pepple, Sarah John, Leslie Wilson, Victoria Wang, Russell N. Van Gelder

**Affiliations:** a University of Washington, Department of Ophthalmology, Seattle, WA, 98104, USA; b University of Washington, Department of Biological Structure, Seattle, WA, 98195, USA; c University of Washington, Department of Laboratory Medicine and Pathology, Seattle, WA, 98195, USA; d University of Washington, Roger and Angie Karalis Johnson Retina Center, Seattle, WA, 98109, USA

**Keywords:** Mycobacterium tuberculosis, Chronic uveitis, Inflammation, Cytokine, IP-10 (CXCL10), IL-17

## Abstract

Post-infectious uveitis describes the condition of chronic immune mediated ocular inflammation associated with pathogens such as Mycobacterium tuberculosis (Mtb). Mtb associated post-infectious uveitis can be modeled in mice by intravitreal injection of heat-killed Mtb (HKMtb). To better understand how prior systemic exposure to the pathogen alters the local immune response to Mtb, we used flow cytometry and multiplex ELISAs to compare ocular responses to intravitreal HKMtb in the presence or absence of a systemic “prime” of HKMtb. Priming resulted in exacerbation of local inflammation with significantly increased clinical and histologic inflammation scores and increased vitreous cytokines concentrations one day after intravitreal injection of HKMtb. Seven days after injection, uveitis in unprimed animals had largely resolved. In contrast in primed animals, clinical signs of chronic inflammation were associated with a significant increase in the number of ocular T cells, NK cells, and Ly6C^hi^ macrophages and increasing vitreous concentrations of IL-17, VEGF, MIG(CXCL9), IP-10(CXCL10), IL-12p40 and MIP-1α(CCL3). In mice lacking mature T and B cells (RAG2 deficient), the impact of priming on the ocular immune response was ameliorated with significantly lower vitreous cytokine concentrations and spontaneous resolution of uveitis. Altogether these results suggest that the ocular response to Mtb is exacerbated by prior systemic Mtb infection and chronic post-infectious uveitis is mediated by local production of cytokines and chemokines that amplify Th17 and Th1 responses. This mouse model of chronic Mtb associated uveitis will help elucidate mechanisms of disease in patients with post-infectious uveitis.

## 1. Introduction

The term ‘uveitis’ describes a heterogeneous set of conditions that all feature intraocular inflammation. Uveitis is relatively common, with an incidence in large, US population-based studies of ~50/100,000 person-years and a prevalence of ~100/100,000 person years (Gritz and Wong, 2004). These numbers suggest that 300,000 individuals in the US currently have active uveitis. Uveitis is highly associated with vision loss, and is the fifth leading cause of blindness in the US and worldwide (Durrani et al., 2004; Nussenblatt, 1990; Rothova et al., 1996; Thorne et al., 2016). Infection causes up to 20% of uveitis in the US and Europe (Shirahama et al., 2018; Bodaghi et al., 2001). In the developing world, almost 50% of cases are thought to be caused by infection (Chang and Wakefield, 2002; Miserocchi et al., 2013). Many pathogens can cause infectious uveitis. The most common pathogens include *Mycobacterium tuberculosis (Mtb)*, *Toxoplasmosis gondii*, *Treponema pallidum*, and viruses such as herpes simplex, varicella zoster, and cytomegalovirus (Lin, 2015). Multiple bacterial and fungal species can also cause infectious uveitis, and new ocular pathogens continue to be identified (Doan et al., 2016; Lee et al., 2015; Varkey et al., 2015).

The mechanisms responsible for uveitis that develops in combination with current or prior evidence of systemic Mtb infection are not well understood (Agrawal et al., 2021; Basu et al., 2020). While direct infection of the eye is one possible mechanism, molecular and histologic investigations have rarely identified evidence of viable infection in inflamed eyes (Wroblewski et al., 2011). As a result, two alternative immune-mediated hypotheses have been proposed (Basu et al., 2020; Wroblewski et al., 2011; Yeh et al., 2012; Tagirasa et al., 2017; Agrawal et al., 2021). The first posits that after effective anti-microbial treatment, components of dead mycobacteria or pathogen associated molecular patterns (PAMPs) can lodge in the eye and stimulate a persistent innate immune response directed against pathogen antigens (Fox et al., 1984; Wakefield et al., 2010; Kumar et al., 2011). The second hypothesis suggests that an inadvertent autoimmune response directed against ocular antigens is generated as a by-product of the appropriate immune response to infection either by antigen mimicry or epitope spread (Wildner and Diedrichs-Mohring, 2003; Thurau et al., 1997; Singh et al., 1989b; Singh et al., 1989a; Tagirasa et al., 2017). Variations on this latter mechanism have also been invoked to explain the pathogenesis behind a range of post-infectious syndromes including Guillain-Barre syndrome, reactive arthritis, and rheumatic heart disease (Ercolini and Miller, 2009).

Previous animal models in guinea pigs and mice have been reported for use in studying Mtb associated uveitis. However, they were limited by low rates of reproducible ocular infection and inflammation following systemic exposure to live Mtb (Rao et al., 2009; Abhishek et al., 2019; Mitchell et al., 2020). To provide an alternative approach, we have utilized heat-killed Mtb (HKMtb) extract in place of live Mtb. To model systemic infection preceding ocular exposure to Mtb, HKMtb is first administered by subcutaneous route seven days prior to intravitreal injection of a small amount (5 μg) of HKMtb. This approach to Mtb associated uveitis induction is termed primed mycobacterial uveitis (PMU) (John et al., 2021). PMU generates an acute panuveitis that slowly resolves into a chronic posterior uveitis over the following month. In the current study, we perform a more comprehensive analysis of the local ocular response to HKMtb and determine the impact of prior systemic exposure to HKMtB antigens on local immune cell and cytokine responses.

## 2. Methods

### 2.1. Animals and uveitis induction

The animal study protocol was approved by the Animal Care and Use Committee of the University of Washington (animal study protocol # 4481–02) and was compliant with the ARVO Statement for the Use of Animals in Ophthalmic and Vision Research. An equal number of male and female mice weighing at least 18 gm and between the ages of 6–10 weeks at the time of uveitis initiation were used in all experiments. Mice used included C57Bl/6J (JAX#000664), B6.Cg-*Rag2*^*tm1.1Cgn*^/J (JAX#008449), B6.129P2(SJL)-*Myd88*^*tm1.1Defr*^/J (JaX#009088). Mice were maintained with standard chow and medicated water (acetaminophen 300 mg/kg) *ad libitum,* with 12 h light: 12 h dark cage lighting under specific pathogen-free conditions. PMU induction in mice is a modification of a protocol previously published in rabbits (Mruthyunjaya et al., 2006; Cousins, 1992; Jaffe et al., 1998) and rats(K. L. Pepple et al., 2015; Kathryn L. Pepple, Wilson, and Van Gelder, 2018) and has been reported previously (Gutowski et al., 2017; John et al., 2020, 2021). Briefly, on day -7 animals receive a subcutaneous injection of 100 μg killed mycobacterium tuberculosis H37Ra antigen (#231141, Difco Laboratories, Detroit, MI) in a 0.1 cc emulsion of incomplete Freund’s adjuvant (#263910, Difco Laboratories, Detroit, MI) divided with half the dose to each hip. Seven days later (designated as day zero) the right eye of each animal receives an intravitreal injection of PBS (sham), 5 μg (low dose), or 10 μg (high dose) of a suspension of killed mycobacterium tuberculosis H37Ra antigen in 1–1.5 μl of phosphate buffered saline (PBS). One cohort of animals had a 28 day interval between subcutaneous and intravitreal injection. They are indicated as P28 PMU in Supplemental Fig. 5. The fellow (left) eye of each animal is an untreated negative control. It is termed unprimed mycobacterial uveitis (UMU) when animals did not receive the subcutaneous priming injection prior to intravitreal injection on day 0.

### 2.2. Optical coherence tomography (OCT) system, image acquisition, and analysis

OCT imaging was performed as described previously (John et al., 2020). Briefly, OCT images were acquired using the Bioptigen Envisu R2300. Anesthesia was provided with 6.9 mg/kg ketamine/xylazine IP (1% solution) (Ketamine: Ketaset 100 mg/mL, Zoeitis, Inc. Kalamazoo, MI; Xylazine: AnaSed 20 mg/mL, Lloyd Laboratories, Shenandoh, IA). Eyes were dilated with phenylephrine (2.5%, Akorn, Inc. Lake Forest, IL) and corneal protection provided by Genteal (Alcon Laboratories, Inc. Fort Worth, TX). For the anterior chamber, a high resolution 3.6 mm vertical B-scan (1000 A-scans/B-scan, with 20 × averaging) of the anterior chamber centered on the pupil was captured using a Bioptigen 12 mm telecentric lens (product # 90-BORE-G3–12, Bioptigen, Inc. Morrisville, NC). For retinal imaging, a high resolution 1.6 mm B-scan centered on the optic nerve (1000 A-scans/B-scan, with 20 × averaging) was captured using the Bioptigen mouse retina lens (product # 90-BORE-G3-M, Bioptigen, Inc. Morrisville, NC). Three masked graders scored OCT images for the degree of inflammation using a 6-step ordinal scale ranging from 0 to +4 (Supplemental Table 4). Final score was determined to be the score assigned by at least 2 of the 3 graders. When media opacity (corneal, lens, vitreous) precluded scoring of an image of the posterior segment, the image was marked as “NA” and the eye was not included in analysis on that day. Close attention to corneal protection during OCT imaging and post-anesthesia recovery is important to prevent corneal damage and poor image quality. For recommendations on corneal protection considerations during OCT imaging see (John et al., 2021).

### 2.3. Histology and immunohistochemistry

Whole eyes were fixed in 10% neutral buffered formalin (Sigma-Aldrich Corp., St. Louis, MO) for 24 h. Paraffin block sections (4 μm) were stained with hematoxylin and eosin (H&E) and scored by a single grader, masked to treatment using the score system in Supplemental Table 5. Immunohistochemistry with DAB secondary antibodies was performed using standard protocols by the University of Washington histology and imaging core facility (www.uwhistologyandimaging.org). Primary antibodies used were rat anti-CD3 (Serotec/clone CD3–12 AbD), rabbit anti-F4/80 (Cell Signaling/clone D2S9R), and rat anti-GR-1 (R&D Systems/clone RB6–8C5). DAB + cells were counted on 3 sections per eye and the average number of DAB + cells/eye was reported for 3 eyes per stain for PMU5 and UMU5 eyes on day 1 and day 56.

### 2.4. Flow cytometry

Tissue from left and right eyes were collected separately for flow analysis as previously described (John et al., 2020). Briefly, eyes were enucleated and conjunctiva removed. Then in a 50 μl pool of PBS with 1% BSA intraocular contents (retina/choroid/aqueous/vitreous/uveal tissue) were collected after removal of lens, sclera, and cornea. The following antibodies were used in this study: Ly6g clone 1A8 AF647 (0.25 ng/μL), Ly6c clone HK1.4 FITC (0.25 ng/μL), CD11b clone M1/70 PerCPCy5.5 (0.04 ng/μL), CD3 clone 17A2 BV421 (1.2 ng/μL), CD19 clone 6D5 BV605 (0.1 ng/μL), NK1.1 clone Pk136 PE (0.2 ng/μL), CD45 clone 30-F11 BUV395 (0.2 ng/μL). Live-dead discrimination was performed using Zombie Aqua. Antibodies were obtained from Biolegend, San Diego, CA, with the exception of CD45-BUV395 which was obtained from BD Biosciences, San Jose, CA. Unstained cell controls were used to determine marker negative cell populations. Samples were fixed overnight at 4 ^◦^C in 1% paraformaldehyde (Electron Microscopy Sciences, Hatfield, PA) in PBS. Compensation was performed using single color controls prepared from BD UltraComp eBeads (Thermofisher, Waltham, MA). Flow cytometric analysis was carried out with an BD LSRII cell sorter (BD Bioscience, Franklin Lakes, NJ, USA). Data analysis was performed using FlowJo v10.1 software (FlowJo LLC, USA). Data presented represents results from at least three independent experiments.

### 2.5. Protein and cytokine analysis

Aqueous humor was collected in an EDTA containing capillary tube (Sarstedt, Nümbrecht, Germany) after corneal paracentesis with a 30-gauge needle (Becton, Dickinson and Company, Franklin Lakes, New Jersey). 1–5 μl of aqueous was collected from each eye, and stored at −80 °C in combination with 1X protease inhibitor (Sigma-Aldrich Corp., St. Louis, MO) until assayed. Aqueous protein was quantified using Pierce 660 nm Protein Assay Reagent (Thermo Scientific, Madison, WI) for colorimetric detection on the Nanodrop ND-1000 spectrophotometer (Thermo Scientific, Madison, WI).

After aqueous collection, the eye was enucleated and frozen on dry ice. The frozen eye was bisected at the limbus. The frozen vitreous was separated from the lens and retina and transferred to 30 μl of 1 X PBS containing 1X protease inhibitor (Sigma-Aldrich Corp., St. Louis, MO). Vitreous of 4 eyes was pooled and stored at −80 °C until assayed. Each pool was tested in triplicate, and the mean value is presented. Serum from all animals was collected by cardiac puncture immediately after death. Serum samples were not pooled. The concentration of 32 cytokines were determined using the Milliplex_MAP_ mouse cytokine/chemokine premixed 32 plex immunology multiplex assay (EMD MilliporeCorp., Billerica, MA). The cytokines measured were Eotaxin, granulocyte-colony stimulating factor (G-CSF), granulocyte monocyte-colony stimulating factor (GM-CSF), IFN-γ, IL-1α, IL-1β, IL-2, IL-3, IL-4, IL-5, IL-6, IL-7, IL-9, IL-10, IL-12 (p40), IL-12 (p70), IL-13, IL-15, IL-17, IFN-γ–induced protein 10 (IP-10), Keratinocyte chemokine (KC), Leukemia inhibitory factor (LIF), lipopolysaccharide-induced CXC chemokine (LIX), monocyte chemoattractant protein-1 (MCP-1), monocyte colony stimulating factor (M-CSF), monokine induced by gamma (MIG), macrophage inflammatory protein-1α (MIP-1α), macrophage inflammatory protein-1β (MIP-1β), MIP-2, vascular endothelial growth factor (VEGF), TNF-α, and regulated on activation, normal T cell expressed and secreted (RANTES). Vitreous pools was diluted (1:1) in RIPA buffer (RIPA, PMSF, PIC) according to the manufacturer’s protocol. Samples were analyzed using the MAGPIX system (Luminex, Austin, TX) with xPonent software version 4.2 (EMD Millipore). Data analysis was performed using Milliplex Analyst Standard Version 5.1 software (EMD Millipore).

### 2.6. Statistical analysis

Statistics analysis and graphing was performed using Graphpad Prism 7.0 software (Graphpad Software, La Jolla, CA.). OCT and histology scores were compared using Kruskal Wallis test with Dunn’s multiple comparisons. Comparison day 1 vitreous cytokine concentrations and flow cytometry results were performed using the Brown-Forsythe and Welch ANOVA with Dunnett’s T3 multiple comparison test. Day 7 UMU and PMU vitreous cytokine concentrations were compared using an unpaired *t*-test with Welch’s correction. P values < 0.05 were considered significant.

## 3. Results

### 3.1. Intravitreal HKMtb generates acute panuveitis that is exacerbated and becomes chronic when preceded by a systemic prime

**Fig. 1.**
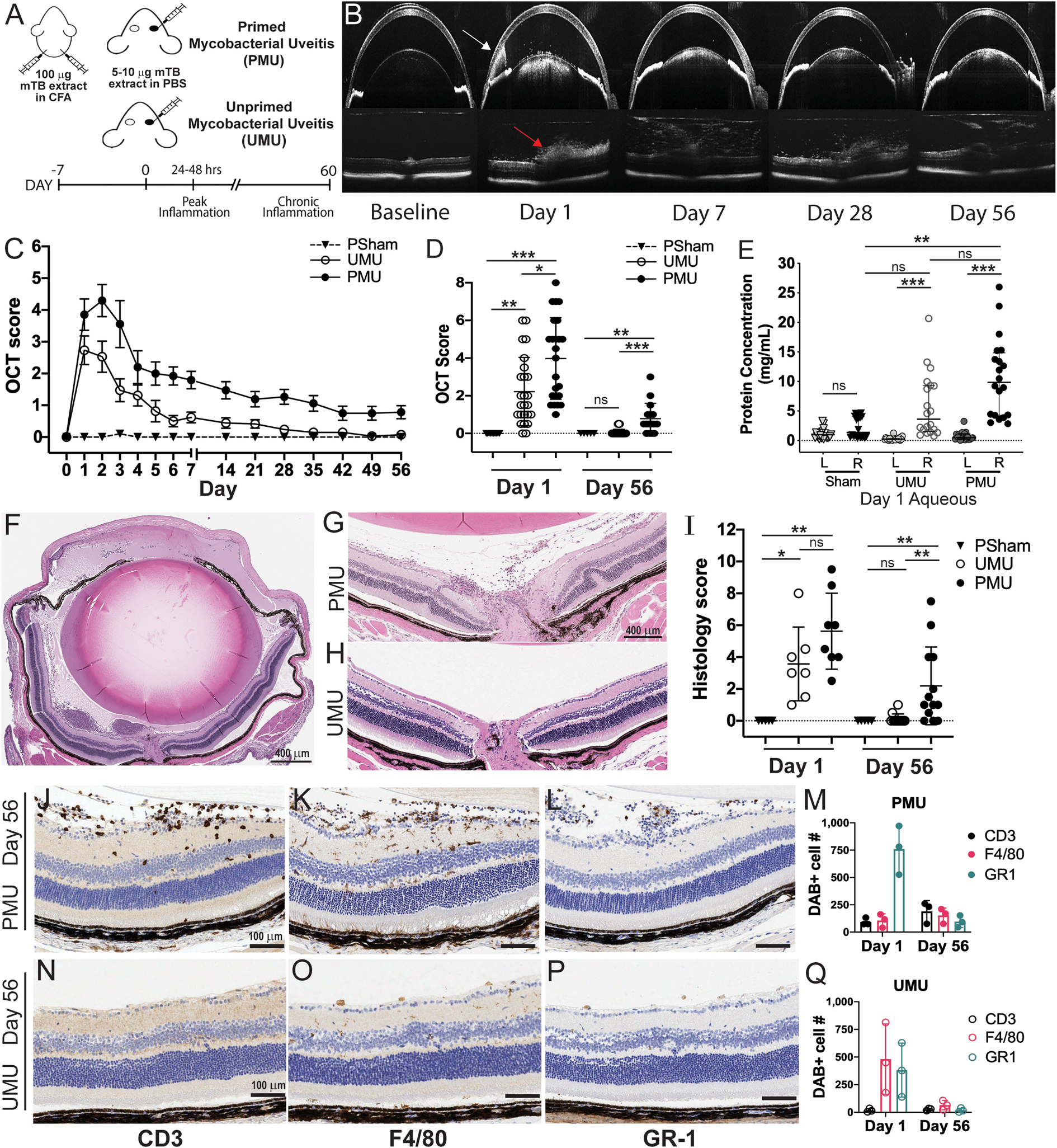
Intravitreal HKMtb generates acute panuveitis that is exacerbated and becomes chronic when preceded by a systemic prime. **(A)** Uveitis is induced with unilateral intravitreal injection of 5 μg heat killed mycobacterial antigen (HKMtb) in PBS with prior systemic CFA prime (PMU) or without the prime (UMU). **(B)** Optical coherence tomography (OCT) imaging is used to monitor inflammation longitudinally. Corneal edema, anterior chamber cells, and hypopyon (white arrow) are visible in anterior images. Posterior chamber images identify vitritis (red arrow) and retinal edema. **(C)** OCT inflammation score by day for PMU n = 17 (black filled circles), UMU n = 17 (open circles), and PBS/Sham injected eyes n = 7 (triangles). Symbol indicates mean score, error is SEM. **(D)** Comparison of day 1 and Day 56 OCT score by treatment condition. Bars indicate the mean and standard deviation. **(E)** Anterior chamber protein concentration from inflamed (R) and fellow (L) eyes. Sham (n = 13), UMU (n = 20), and PMU (n = 20) animals. Bars indicate the mean and standard deviation. **(F)** H&E staining of a day 1 PMU eye. **(G)** Day 56 histology of PMU eye with vitritis, perivascular leukocytes, and a retinal fold. **(H)** Day 56 UMU histology with no inflammation. **(I)** Comparison of histology scores between sham injected (triangle), UMU (open circles), and PMU (closed circles) animals on day 1 and day 56 (n = 5–13/condition). **(J**–**L)** Day 56 PMU retina with DAB+ **(J)** T cells (CD3^+^), **(K)** macrophages and retinal microglia (F4/80+) and **(L)** neutrophils (Gr-1+). **(M)** Quantification of DAB + cells on days 1 and day 56 in PMU, CD3 (black), F4/80 (pink), or GR1 (cyan). **(N–P)** Day 56 UMU retina with **(N)** rare T cells (CD3^+^), **(O)** few macrophages and retinal microglia (F4/80+) and **(P)** no neutrophils (GR-1+)**. (Q)** Quantification of DAB + cells on days 1 and day 56 in UMU, CD3 (black), F4/80 (pink), or GR1 (cyan). Comparisons of OCT score, anterior chamber protein concentration, and histology score performed by day with Kruskal Wallis test with Dunn’s multiple comparisons.*p < 0.5, **p < 0.01, ***p < 0.001. (For interpretation of the references to color in this figure legend, the reader is referred to the Web version of this article.)

**Fig. 2.**
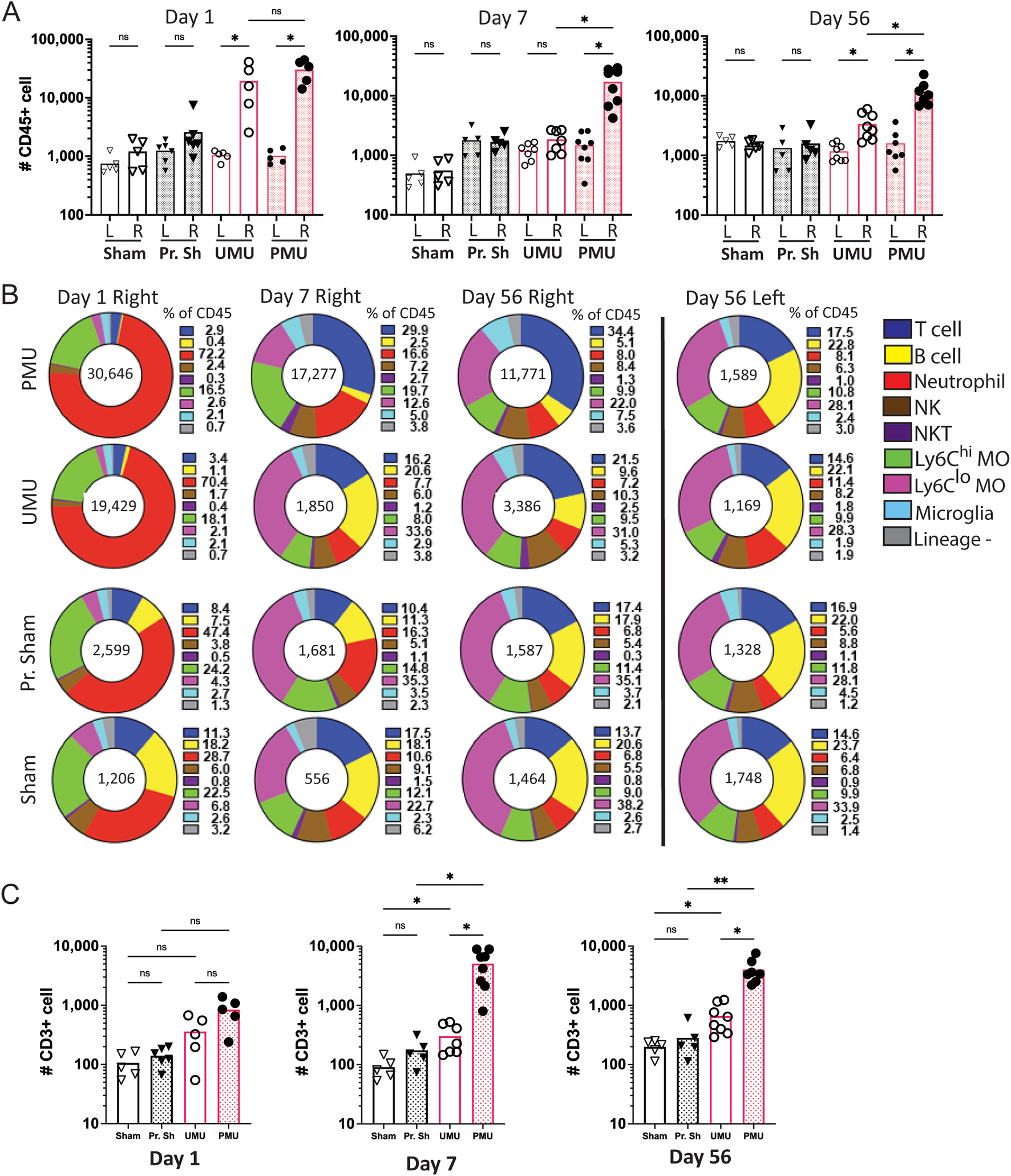
Priming leads to significantly increased total ocular CD45^þ^ cells and T cells by day 7 after IVT. **(A)** Ocular CD45^+^ cell number for injected right (R) and control left (L) eyes on days 1, 7 and 56 after intravitreal injection. Each point represents the cell count from an individual eye. Sham (Sham), primed sham (Pr. Sh), unprimed Mtb (UMU), primed Mtb (PMU). All comparisons indicated. Significance determined by Brown-Forsythe ANOVA with Dunnett’s T3 multiple comparison test. **(B)** Pie charts show the distribution of inflammatory cell types present as the percentage of CD45^+^ cells. Average number of CD45^+^ shown in the center of the pie chart. Neutrophils are the dominant population on day 1. T cells (dark blue) become the major cell population in PMU eyes on day 7. In contrast CD11b^hi^, Ly6C^lo^ monocytes (pink) become the major population in UMU eyes. **(C)** Total number of ocular T cells on days 1, 7 and 56. PMU eyes have significantly more T cells than UMU eyes on days 7 and 56. Unpaired *t*-test with Welch’s correction. ns = not significant, *p < 0.05. NK = natural killer cell, NKT = natural killer T cell, Ly6C^hi^ MΦ (macrophages), Ly6C^lo^ MΦ (macrophages). (For interpretation of the references to color in this figure legend, the reader is referred to the Web version of this article.)

**Fig. 3.**
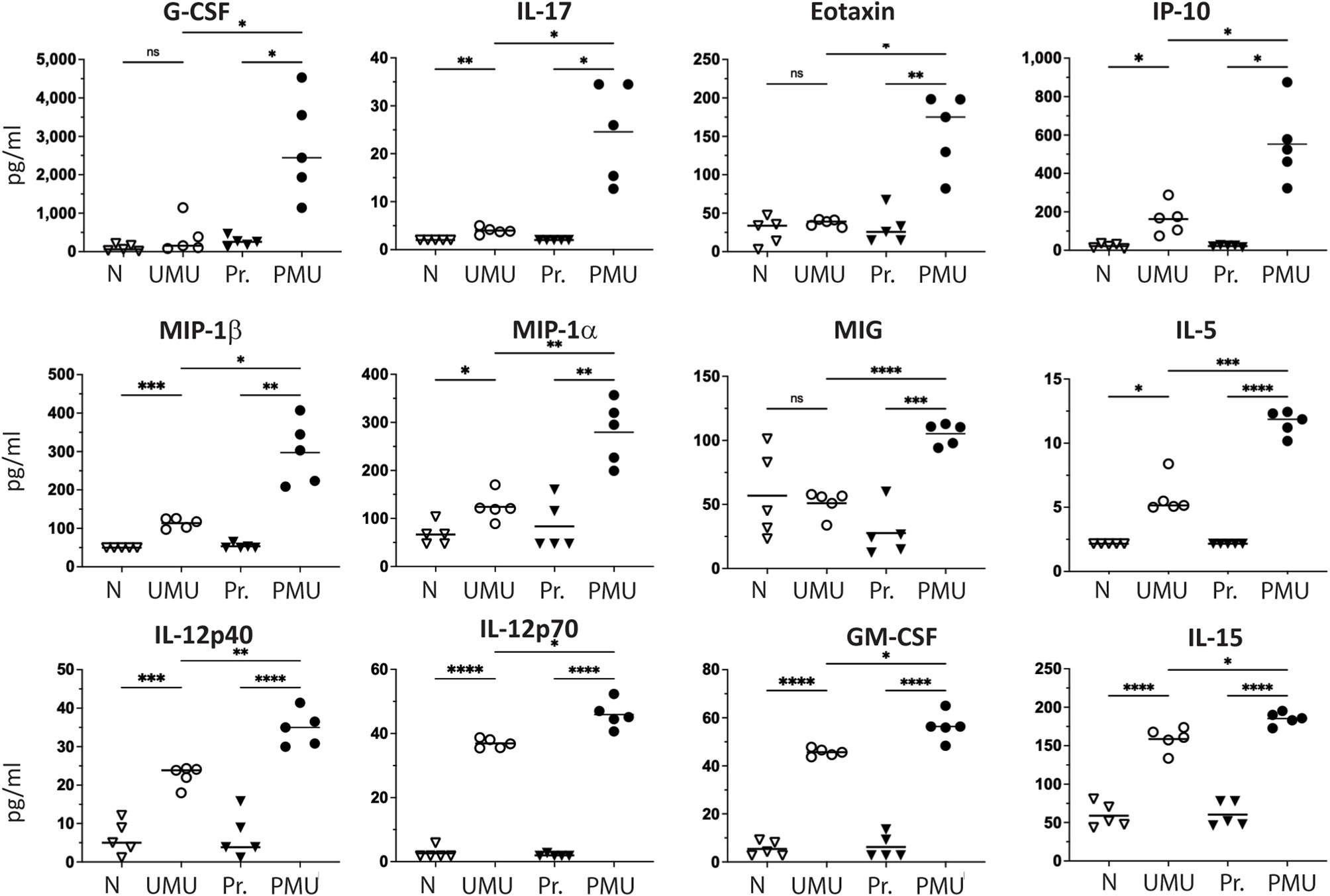
Priming significantly increases vitreous cytokine concentrations on day 1 after intravitreal injection of HKMtb extract. Twelve cytokines were significantly increased in PMU vitreous when compared to UMU. Bar indicates mean concentration of all samples per condition (n = 5). Cytokine concentrations were also elevated when compared to control vitreous from naive (N) or primed naive (Pr) animals. Significant differences determined with Brown-Forsythe ANOVA with Dunnett’s T3 multiple comparison test. ns = not significant, *p < 0.05, **p < 0.01, ***p < 0.001, ****p < 0.0001.

To better understand the ocular response to successfully treated Mtb infection, an extract of heat killed Mtb was injected into the vitreous of the right eye of C57BL/6 mice and the resulting uveitis was assessed using optical coherence tomography (OCT) imaging, histology (Fig. 1), flow cytometry (Fig. 2) and vitreous cytokine concentration measurement (Fig. 3). Additionally, to determine the impact of prior systemic exposure to Mtb before ocular exposure (as occurs in post-infectious uveitis) these results were compared to the ocular responses generated by animals that had received a systemic antigen prime seven days before ocular injection. These two conditions were termed unprimed mycobacterial uveitis (UMU) and primed mycobacterial uveitis (PMU) (Fig. 1A). After intravitreal injection of HKMtb, both unprimed and primed eyes demonstrated acute inflammation with the presence of anterior chamber cell, hypopyon, pupillary membrane, and vitritis (Fig. 1B,F). In both conditions, inflammation was detectable as early as 10 h after intravitreal injection by OCT (data not shown). In unprimed animals, OCT inflammation score peaked 24 h after intravitreal injection (2.7 ± 1.9). When compared on day 1, primed animals had significantly higher OCT inflammation score (3.9 ± 2.0, p < 0.05) than unprimed animals (Fig. 1C and D). In primed animals, inflammation peaked 48 h after injection with a score of 4.3 ± 2.1.

Uveitis in humans is also associated with an increase in aqueous protein concentration caused by breakdown of the blood aqueous barrier (Shah et al., 1992). To determine the impact of HKMtb intravitreal injection on aqueous flare, protein concentration was measured one day after intravitreal injection and compared to sham injection and fellow eye controls (Fig. 1E). Aqueous protein in HKMtb injected eyes on day 1 was significantly increased in both unprimed and primed animals when compared to fellow eye controls(P < 0.001), but only the aqueous from primed animals was significantly higher than sham injected aqueous (p < 0.01).

Using longitudinal OCT imaging to monitor signs of inflammation in a subset of animals over time, we found that inflammation scores decreased between days 2 and 7 in both primed and unprimed animals (Fig. 1C). However, in the primed animals, OCT scores remained elevated (above 1) for one month while unprimed OCT scores fell to baseline (score 0–0.5) between 2 and 4 weeks after intravitreal injection. Signs of chronic inflammation seen on OCT in primed eyes included persistent diffuse vitritis, focal hyperreflective deposits near retinal vessels, and rare outer retinal damage including retinal folds (Fig. 1B). On day 56, the final OCT score of 0.8 ± 0.7 in primed eyes was significantly elevated compared to baseline score prior to intravitreal injection in the same animals (p < 0.001) and to the day 56 scores in unprimed (0.1 ± 0.2) and sham injection controls (p < 0.01) (Fig. 1C and D).

Histology of both primed eyes (Fig. 1F) and unprimed eyes on day 1 after intravitreal injection demonstrated marked leukocyte infiltration into the vitreous and aqueous. When scored for inflammation severity on day 1, eyes from primed animals were on average more inflamed than eyes from unprimed animals, but the difference was not significant (Fig. 1I). Histology performed on eyes collected 56 days after intravitreal injection reflected the marked difference in inflammation associated with priming previously noted by OCT (Fig. 1G and H). Eyes from primed showed persistent inflammatory cells in the vitreous as well as many perivascular inflammatory cells, and rare outer retinal folds (Fig. 1G) In contrast eyes from unprimed animals were not inflamed (Fig. 1H).

To characterize the types of inflammatory cells present in HKMtb injected eyes, immunohistochemistry on whole eye sections was performed to detect T-cells (CD3^+^), macrophages (F4/80+), and granulocytes (Gr-1+) and cells were counted on serial sections for days 1 and 56 (Fig. 1J–Q). The acute (day 1) inflammatory infiltrate was identified as predominantly Gr-1+ granulocytes and F4/80+ macrophages located primarily in the aqueous and vitreous (Supplemental Fig. 1 and Figure 1M). On day 56, eyes from primed animals demonstrated CD3^+^ cells infiltrating the retina (Fig. 1J) as well as numerous F4/80 (Fig. 1K) and GR-1+ cells (Figure 1L) in the vitreous. In contrast, few inflammatory cells were detected in unprimed eyes (Fig. 1 N–Q and Supplemental Fig. 1). Together these data indicate that intravitreal HKMtb generates acute panuveitis that is exacerbated and becomes chronic when preceded by a systemic prime.

### 3.2. Priming significantly increases ocular CD45^+^ cell and T cell number 7 days after intravitreal injection of heat killed Mtb

Flow cytometry was then used to measure and characterize the major leukocyte populations infiltrating the eye following HKMtb injection in primed and unprimed animals. As a measure of inflammation severity, the total number of CD45^+^ cells/eye was compared between primed and unprimed animals injected with HKMtb or PBS (sham) injected controls. Sham injection did not significantly increase ocular CD45^+^ cell number when compared to uninjected fellow eyes at any time point, but higher numbers of neutrophils were detected on days 1 and 7 indicating sham injection does generate transient, low-level, sterile inflammation (Supplemental Fig. 3). In contrast, eyes injected with HKMtb demonstrated significantly more CD45^+^ cells/eye when compared to fellow uninjected eyes and PBS (sham) injection controls (Fig. 2A). When eyes receiving HKMtb injection were compared, priming led to a higher average number of CD45^+^ cells/eye, on all days but the differences were only significant (p < 0.05) at the later time points (Fig. 2A and B).

To determine if priming changed the types of cells infiltrating the eye after HKMtb injection, ocular CD45^+^ cells were classified as neutrophils, Ly6C^hi^ or Ly6C^lo^ macrophages, NK cells, NKT cells, T cells, B cells, presumed microglia (CD11b^lo^) or lineage negative cells using a multicolor flow cytometry staining approach (see gating strategy in Supplemental Fig. 2). On days 1,7, and 56 these populations were evaluated as both a % of all ocular 45+ cells (Fig. 2B) and for the total number of each cell type (Supplemental Fig. 3).

On day 1, the majority of CD45^+^ cells in the eye were neutrophils (shown in red in Fig. 2), and there were no significant differences in the number of each cell type present in eyes injected with HKMtb based on the presence of the systemic prime (Supplemental Fig. 3). However, by day 7 differences in ocular cell CD45^+^ populations developed based on the presence of the prime. Primed eyes developed a T cell dominant infiltrate (29.9% of CD45^+^ cells) while unprimed eyes were primarily invaded by Ly6Clo macrophages (33.6% of CD45^+^ cells. When total cell number was compared, eyes of primed animals injected with HKMtb had significantly more T cells (Fig. 3C), NK cells, and Ly6C^hi^ macrophages (Supplemental Fig. 3) than unprimed eyes as chronic inflammation began to develop by day 7 and continued through day 56. In primed eyes, T cells (in blue) became the dominant CD45^+^ population on day 7 and continued through day 56 averaging between four and six thousand cells per eye (Fig. 2C). These data indicate that chronic uveitis in PMU is associated with a local infiltration of T cells that begins 7 days after ocular injection of HKMtb. Similar results were obtained when these experiments were repeated using an increased dose of intravitreal HKMtb (10 μg), with the exception that the uveitis in unprimed animals did not resolve spontaneously by day 56 (Supplemental Fig. 4). Extending the time between the systemic prime and the intravitreal injection from 7 to 28 days did not change OCT scores, CD45^+^ or CD3^+^ cell numbers confirming that the presence or absence of the prime rather than the timing of the prime is the significant variable in the development of chronic uveitis (Supplemental Fig. 5). B cells are another key effector cell of the adaptive immune system. While the ocular B cell percentage varied between conditions (0.4–23.7% of CD45^+^ cells/eye), the average number of total B cells per eye was small and ranged from 244 to 354 cells/eye (Supplemental Fig. 3). This suggests that these B-cells represent the number present in blood circulating through the eye at the time of collection rather than a disease associated ocular B cell population.

### 3.3. Priming significantly increases vitreous cytokine concentrations in response to intravitreal HKMtb

Cytokines and chemokines are important mediators of ocular inflammation, and the presence of certain cytokines in an inflamed tissue can suggest specific pathogenic mechanisms. Due to the different outcomes noted in the unprimed and primed eyes injected with 5 μg HKMtb extract (spontaneously resolving vs chronic uveitis), we hypothesized that there would be significant difference in the ocular cytokine profiles from inflamed eyes of each model. To test this hypothesis, the concentrations of 32 vitreous cytokines were measured on day 1 and day 7 after intravitreal injection.

To determine the baseline concentrations in an uninflamed eye, vitreous of uninjected healthy control eyes (naive) as well as uninjected eyes of animals that had received a subcutaneous prime (primed naive) were evaluated. In both conditions, cytokine concentrations were low or below the level of detection by the assay and there were no significant differences in measurable cytokine concentrations between the naive and primed naive controls (Supplemental Table 1).

Next vitreous cytokines were measured from UMU and PMU eyes 1 day after intravitreal injection of 5 μg of HKMtb extract. When UMU vitreous was compared to naive controls, 21 of the 32 cytokines measured (66%) were significantly elevated. In PMU vitreous, 27 cytokines (84%) were significantly increased when compared to primed naive controls. Overall, cytokine concentrations were higher in PMU eyes than in UMU vitreous (1.2–7.3 fold higher) and twelve of the 32 cytokines (38%) were significantly higher. The cytokine significantly elevated in PMU vitreous when compared to UMU vitreous included G-CSF, IL-17, Eotaxin, IP-10, Mip-1β, Mip-1α, MIG, IL-5, IL-12p40, IL-12p70, GM-CSF, and IL-15 (Fig. 3). These findings are consistent with the previous observation that PMU eyes demonstrate more severe acute OCT inflammation scores than UMU eyes. The only cytokines that were found in higher concentration in UMU vitreous when compared to PMU vitreous were IL-9 and IL-13, but these differences were not significant.

### 3.4. Chronic inflammation in HKMtb injected eyes is associated with increasing concentrations of IL-17, VEGF, MIG, IP-10, IL-12p40 and MIP-1a

On day 7 after intravitreal injection the clinical courses in unprimed and primed animals diverge with uveitis in UMU eyes spontaneously resolving while PMU eyes develop chronic uveitis. Therefore to further characterize and compare the cytokines associated with these different outcomes, vitreous concentrations were determined from day 7 PMU and UMU eyes and compared. Consistent with higher clinical inflammation scores in PMU on day 7, the majority of cytokines (22/32 or 69%) were found in significantly higher concentration in PMU day 7 vitreous when compared to UMU day 7 vitreous (Supplemental Table 2). This difference was in large part due to the fact that the majority of cytokines in day 7 UMU vitreous had returned to near baseline control concentrations. Only 4 cytokines in UMU day 7 vitreous remained elevated at least 2 fold above baseline vitreous concentrations including IP-10, IL-6, KC, and G-CSF. However, only IP-10 was significantly increased on Day 7 when compared to baseline naive vitreous(92 ± 39 pg/ml vs. 22 ± 11 pg/ml, p = 0.032) (Supplemental Fig. 6).

**Fig. 4.**
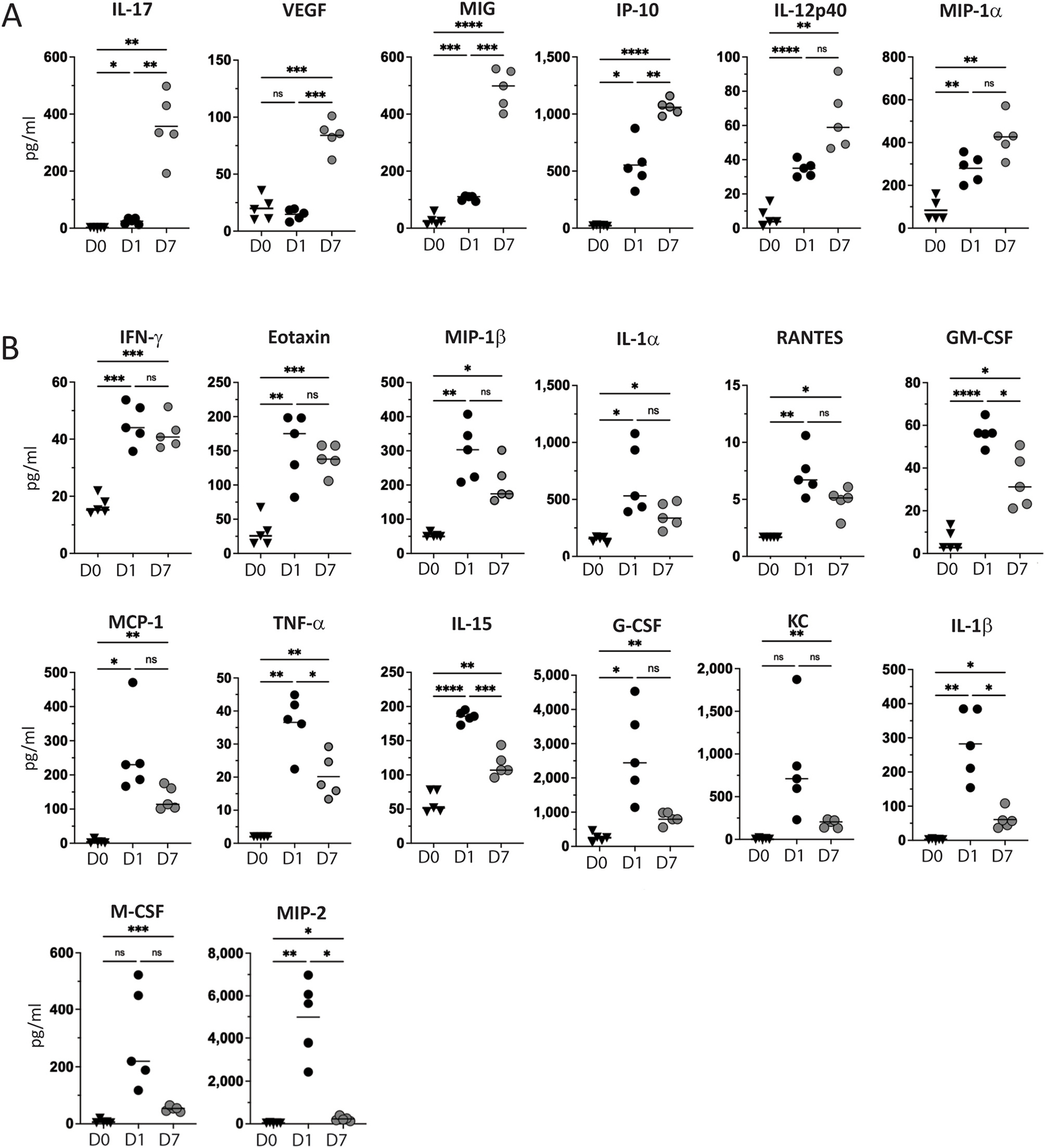
In primed animals, many vitreous cytokines remain elevated or increase seven days after HKMtb injection. Vitreous cytokines were measured on day 7 after IVT HKMtb and compared to primed naive (D0) and day 1 (D1) results. **(A)** Six cytokines demonstrated increased concentrations on D7. **(B)** Fourteen cytokines had decreased when compared to D1, but remained significantly elevated on D7 when compared to D0. Significant differences determined with Brown-Forsythe ANOVA with Dunnett’s T3 multiple comparison test. ns = not significant, *p < 0.05, **p < 0.01, ***p < 0.001, ****p < 0.0001.

In contrast, in PMU day 7 vitreous, 25 cytokines remained >2 fold higher than baseline concentrations, of which 20 were significantly higher (Fig. 4A and B). When the longitudinal concentrations of these cytokines were evaluated, two patterns emerged. Six cytokines showed increased concentration between day 1 and day 7 (Fig. 4A) including IL-17, VEGF, MIG, IP-10, IL-12p40 and MIP-1α. Interestingly, the 15-fold increase in IL-17 concentrations from day 1 (24.6 ± 10.3 pg/ml) to day 7 (357 ± 116 pg/ml) indicates chronic uveitis in PMU is likely mediated by a Th17 dependent mechanism. The remaining fourteen cytokines that remained increased when compared to baseline were decreased in concentration on day 7 when compared to day 1. Some, like IFN-gamma, Eotaxin, MIP-1B, IL-1alpha, and RANTES showed minimal or non-significant decreases on day 7 (Fig. 4B) suggesting they may play a role in both acute and chronic inflammation. However, many had decreased significantly by day 7 indicating that they were likely returning to baseline levels and functioned primarily in acute inflammation. Taken together, these cytokine results identify an acute intraocular proinflammatory cytokine profile associated with acute post-infectious uveitis, and highlights the impact of prior systemic priming on the expression of ocular inflammatory cytokines. Furthermore, the longitudinal data from PMU vitreous on days 1 and 7 identifies on-going inflammatory cytokine expression in primed animals and highlights a subset of cytokines that may be mechanistically important in the pathogenesis of chronic post-infectious uveitis.

Serum cytokine concentrations were also measured to determine how priming impacted systemic measures of inflammation and to ensure vitreous cytokine levels did not result from passive transfer across the blood ocular barrier (Supplemental Table 3). In serum collected from animals prior to intravitreal injection (baseline), less than half (14/32) of the cytokines were detectable in either primed or unprimed animals. Of those detected, priming significantly increased the serum concentrations of G-CSF, IL-1α, IL-9, IP-10, and MIP-1α (Supplemental Fig. 7A). When serum concentrations in primed animals were compared to vitreous concentrations on day 1 after HKMtb injection, local (ocular) cytokine concentrations were higher than serum concentrations for 30/32 cytokines tested. Higher ocular concentrations confirm local production of these cytokines. The two exceptions were Eotaxin and MIG which were notable for higher concentrations in the serum than in the vitreous suggesting that vitreous levels may be impacted by serum levels following inflammation induced blood ocular barrier disruption (Supplemental Fig. 7B). When vitreous and serum concentrations were compared again on day 7, almost all cytokines continued with higher concentrations within the eye than in the serum. Of particular interest were IL-17, VEGF, MIG, IP-10, IL-12p40 and MIP-1α, all cytokines noted to increase over time in the vitreous. When compared to day 7 serum levels, vitreous concentrations were often 5–10 fold higher (Supplemental Fig. 7C) supporting their importance as local mediators of chronic uveitis. In contrast, three cytokines were found in higher concentration in the serum of primed animals than in the vitreous on day 7 including G-CSF, Eotaxin, and IL-1α suggesting that ocular levels likely reflect continued blood ocular barrier permeability rather than local production (Supplemental Fig. 7D).

### 3.5. Adaptive immunity is necessary for increased uveitis severity and chronicity in primed animals

**Fig. 5.**
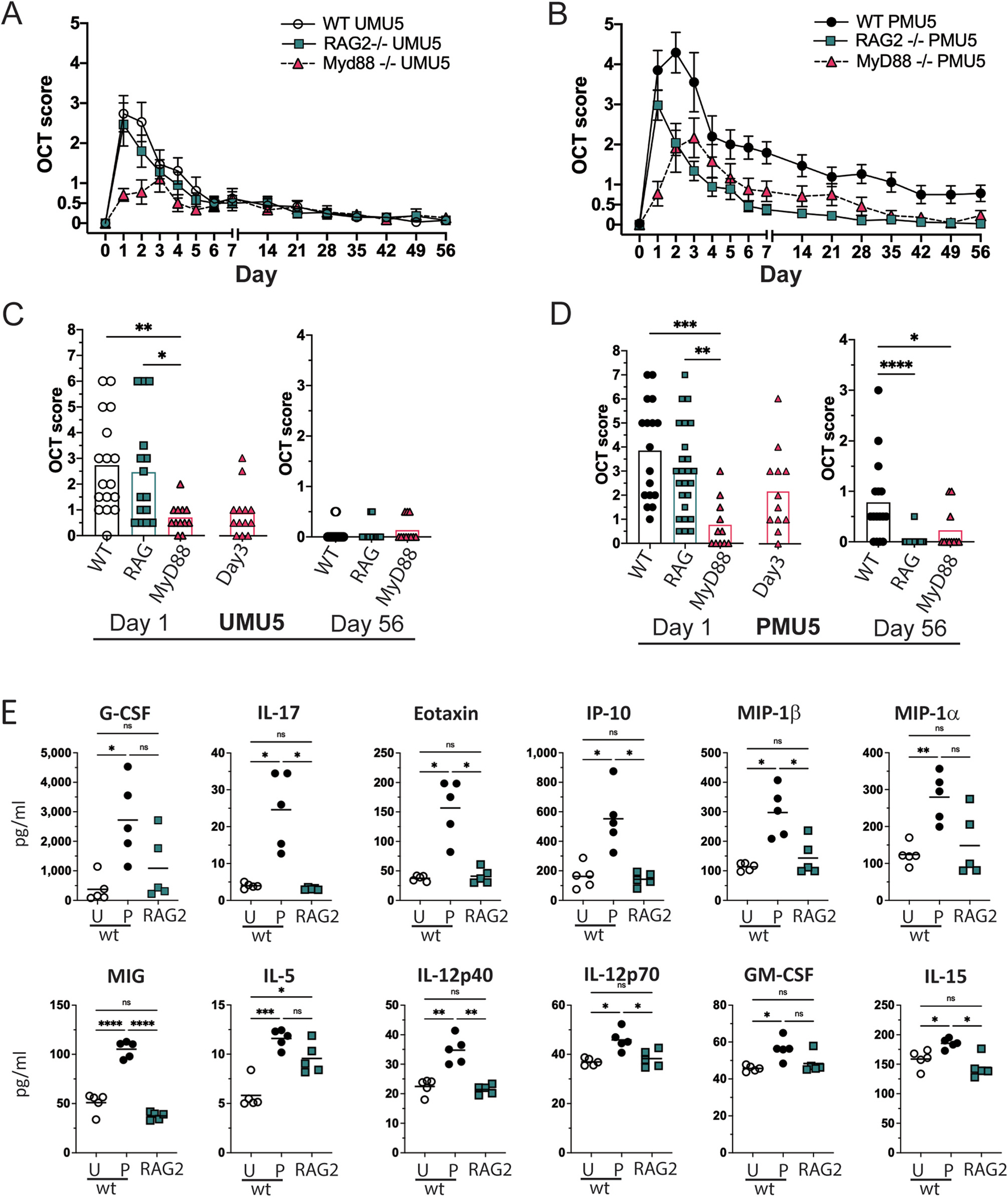
Innate immunity is necessary for uveitis in unprimed animals while adaptive immunity is necessary for increased uveitis severity and duration in primed animals. OCT inflammation score was compared in wild type, RAG-2 deficient, and MyD88 deficient animals in the primed **(A,C)** and unprimed **(B, D)** models. Symbols for wild type animals shown in black and white, RAG-2 deficient indicated by cyan squares, and MyD88 deficient indicated by pink triangles. Bar indicates mean score. Comparison performed with Kruskal Wallis test with Dunn’s multiple comparisons. (E) In wild type animals, twelve vitreous cytokines were significantly increased in primed (P) compared to unprimed (P) animals on day 1. In primed RAG-2 animals, vitreous cytokines were decreased when compared to wild-type primed animals, and not significantly different when compared to unprimed wild-type animals. *p < 0.5. **p < 0.01, ***p < 0.001,****p < 0.0001. (For interpretation of the references to color in this figure legend, the reader is referred to the Web version of this article.)

The presence of the large T cell population in the eyes of primed animals suggests that an antigen specific adaptive immune response to the intravitreal HKMtb may be responsible for the development of chronic inflammation. To test the hypothesis that the prime effect is dependent on a functional adaptive immune response, RAG-2 deficient animals (RAG-2), which lack functional B and T cells (Shinkai et al., 1992), were given an intravitreal injection of HKMtb extract with or without the systemic prime and OCT scores over time were compared to wild type controls (Fig. 5A–D). As predicted, primed RAG-2 animals did not develop chronic inflammation by OCT or histology, and day 56 OCT and histology scores were significantly lower in primed RAG-2 animals when compared to wild type controls (Fig. 5D and data not shown). Furthermore, the concentration of all vitreous cytokines from primed RAG-2 animals were decreased when compared to wild type day 1 PMU results (Supplemental Table 1). Importantly, 9 of the 12 cytokines that were previously identified as significantly increased on day 1 in primed eyes when compared to unprimed eyes, were significantly decreased in RAG-2 vitreous (Fig. 5E). Furthermore, the concentrations of cytokines in RAG-2 vitreous were similar to concentrations in the wild the unprimed vitreous. Together these data support the hypothesis that the impact of priming on disease severity and differential vitreous cytokine concentrations in PMU is mediated by adaptive immunity.

The presence of acute inflammation in the RAG deficient animals suggests that innate immune function is sufficient to detect intraocular HKMtb extract and initiate acute uveitis. Mycobacterial components are known TLR2 and TLR4 agonists (Su et al., 2005), and MyD88 dependent signaling is necessary to prevent lethality in response to infection to tuberculosis (Cervantes, 2017; Ishikawa et al., 2017; Means et al., 1999). Therefore, in the absence of MyD88, we would expect decreased ability of ocular immune cells to sense the presence of intravitreal HKMtb extract leading to attenuated acute inflammation in the primed and unprimed models. As predicted, MyD88 deficiency significantly decreases acute OCT inflammation score on day 1 in both primed (p < 0.001) and unprimed eyes (p < 0.01) (Fig. 5 A,C). However, MyD88 was not necessary for inflammation in primed animals (Fig. 5 B,D) as these eyes demonstrated an OCT score of 2.2 ± 1.7 on day 3 after intravitreal injection. In contrast in unprimed animals (Fig. 5 A,C), acute inflammation was attenuated on all days following intravitreal injection with a maximum score of only 1.0 ± 0.9 on day 3.

## 4. Discussion

In this study, we found that systemic priming significantly exacerbates the ocular inflammatory response to intravitreal HKMtb. Priming led to increases in vitreous cytokine concentrations, a significant ocular T cell population, and the development of chronic uveitis. Additionally, the chronic uveitis induced by priming is associated with a significant increase in the expression of the Th1 and Th17 associated cytokines and chemokines that are known to amplify and sustain inflammation in other chronic autoimmune conditions. In contrast, in the absence of prior exposure to Mtb (unprimed animals), chronic uveitis does not develop and vitreous cytokine concentrations return to near baseline within 1 week of intravitreal injection.

The comparison performed here between primed and unprimed mice allows for evaluation of the contribution of the systemic immune state (primed versus unprimed) to local inflammation in the eye. However, only the results obtained in primed animals are likely to be relevant to the human condition of Mtb associated uveitis due to the fact that in humans, ocular exposure to Mtb only occurs after systemic infection (with rare exception of trauma-associated direct inoculation). The antigen specificity of the adaptive immune response remains an important question. In mice primed with HKMtb, a strong antibody response is generated against this killed mycobacterial extract (data not shown). However, in human patients with Mtb associated uveitis a population of anti-ocular T cells has also been reported (Tagirasa et al., 2017). Whether the T-cells present in mice eyes recognize the same or different antigens as seen in human eyes with Mtb associate uveitis remains to be determined. However, the unprimed results demonstrate that even in the absence of a pre-existing adaptive immune response to Mtb, the eye can still respond to Mtb PAMPs through MyD88 dependent innate signaling and generate a robust panuveitis with significantly increase concentrations of many pro-inflammation cytokines. In healthy vitreous, inflammatory cytokine concentrations are low or undetectable and there are redundant mechanisms that work to dampen inflammation and maintain homeostasis when innate stimuli are encountered. Further mechanistic studies will be needed to determine what aspects of the unprimed ocular microenvironment allows eyes to escape the fate of chronic inflammation.

In this study, many of the inflammatory cytokines that would be expected as part of an innate response to Mtb such as Il-6, IL-1α and Il-1β and TNF-α, and were found in the vitreous, and that in primed vitreous the concentrations were generally higher than in unprimed vitreous (Paik et al., 2020; Cooper et al., 2011). In human eyes with presumed TB associated uveitis, and chronic idiopathic uveitis, many of the same cytokines that were identified in this study including IP-10, IL-12, VEGF, MCP-1, MIP-1α, and MIP-1β have been identified (Errera et al., 2022; Curnow et al., 2005; El-Asrar et al., 2011; Xu et al., 2020; Nagata et al., 2012; Chen et al., 2015; Zhao et al., 2015; Fukunaga et al., 2020). Cross sectional studies in human eyes typically analyze eyes with different forms of uveitis and stages of disease activity or chronicity which may make interpretation of results regarding the importance of specific cytokines challenging. One strength of our study is the longitudinal collection of ocular samples which provides a link between cytokine concentration changes and the distinct outcomes of chronic inflammation and spontaneous resolution.

Between day 1 and day 7, clinical inflammation in both primed and unprimed animals decreased or nearly resolved, respectively. We therefore expected vitreous inflammatory cytokine concentrations would also decrease. Unprimed vitreous performed as expected with all cytokine concentrations returning to baseline or markedly decreased on day 7 except IP-10 (CXCL10) which was the only cytokine that remained significantly elevated above baseline in unprimed vitreous (Supplemental Fig. 4). In primed eyes, most cytokines also decreased in concentration, however a subset increased in concentration when compared to day 1 including IL-17, MIG (CXCL9), IP-10 (CXCL10), VEGF, IL-12p40 and Mip-1α. This unique pattern of longitudinal expression suggests they may play a significant role in orchestrating the decision between spontaneous resolution and chronic inflammation after an eye is exposed to Mtb.

IL-17 is well established as a central effector cytokine for ocular inflammation in experimental autoimmune uveitis (EAU) as well as in many other chronic autoimmune diseases (Luger et al., 2008; Peng et al., 2007; McGeachy et al., 2019). IL-17 can be produced by a wide range of cells, is a potent chemotactic signal for neutrophils, and is an important cytokine for polarizing local dendritic cells to promote Th17 differentiation. In the day 1 acutely inflamed eyes, neutrophils are likely a main source of the elevated IL-17 as well as many of the other cytokines present in high concentrations such as MIP-2, IL-6. However, over time, as the neutrophil population in the eye declines, IL-17 production likely shifts to T-cells. In patients with Mtb associated uveitis vitrectomy samples have identified CD4^+^ T cells expressing IL-17 and IFN-γ (Sharma et al., 2018; Tagirasa et al., 2017). Future studies will be required to determine if interruption of IL-17 locally or systemically would be effective in treating chronic Mtb associated uveitis.

Like IL-17, IFN-γ was significantly increased in HKMtb injected eyes on day 1 when compared to baseline controls. However, unlike IL-17 which increased more than 10 fold between days 1 and 7 (from 24 pg/ml to 357 pg/ml), IFN-γ concentrations did not increase with time (from 45 pg/ml to 42 pg/ml). Despite this, there was evidence of robust local amplification of the IFN-γ signal with a four fold increases in MIG (from 105 pg/ml to 489 pg/ml) and IP-10 (from 552 pg/ml to 1060 pg/ml) between days 1 and 7 in the vitreous of primed animals. Monokine induced by gamma (MIG), or CXCL9, and interferon gamma induced protein 10 (IP-10) or CXCl10 are both chemokines induced by IFN-γ signaling that are important in the recruitment of activated T-cells to sites of infection and act as local inflammation amplification signals through interaction with their receptor CXCR3 (Park et al., 2002; Lacotte et al., 2009). Both CXCL9 and CXCL10 can be secreted by many cell types in the central nervous system, including tissue resident microglia, astrocytes, inflammatory macrophages recruited from the peripheral circulation, the RPE and Muller glia (Brice et al., 2001; Juel, 2012). We and others have previously identified CXCL10 in ocular samples of rats with PMU and EAU (K. L. Pepple et al., 2015) and it has been identified in samples of from human eyes with autoimmune and infectious forms of uveitis (Norose et al., 2011; Errera et al., 2022), suggesting this chemokine is important chemoattractant in many forms of chronic ocular inflammation. IP-10 was also the only chemokine that remained significantly elevated when compared to baseline in unprimed vitreous on day seven suggesting it is the most resistant to tolerizing influences in the ocular microenvironment. Systemic depletion of this chemokine using biologic therapy has proved effective in controlling inflammation in animal models of myositis and type 1 diabetes, but exacerbated disease in a model of EAE (Christen et al., 2003; Narumi et al., 2002; Kim et al., 2014). Our data suggests local or systemic modulation of IP-10 should be explored further as a potential target for use in controlling chronic ocular inflammation.

In the current study, VEGF concentrations were not significantly increased on day 1 during acute inflammation in either the unprimed or primed vitreous. However, on day 7, VEGF levels were significantly increased in the primed and now chronically inflamed vitreous. VEGF is a dimeric glycoprotein that induces the proliferation and migration of endothelial cells to form new blood vessels, and has additional proinflammatory functions mediated through its effects on endothelial cells. VEGF increases endothelial cell permeability (Senger et al., 1990), endothelial expression of cell adhesion molecules and chemokines (Reinders et al., 2003). Increased concentrations of VEGF have also been reported from EAU retinas that lacked neovascularization suggesting that VEGF functioned primarily to promote autoimmune inflammation in these eyes (Vinores et al., 1998). A previous study in a model of allograft rejection showed that depletion of VEGF had a potent anti-inflammatory effect with inhibition of leukocyte infiltration (CD68^+^ monocytes and CD3^+^ T cells) and endothelial cell chemokine expression that was independent of an impact on angiogenesis (Reinders et al., 2003). Intravitreal Anti-VEGF has also demonstrated anecdotal evidence of clinical benefit in treating eyes with uveitic macular edema (Weiss et al., 2009). Together with our data, these results would suggest that local anti-VEGF therapy should be explored for potential benefit in a wider array of manifestations of chronic uveitis.

Recovery of ocular homeostasis after a uveitis flare occurs spontaneously in some patients, but currently it is not possible to predict which patients will have this desired outcome. Experimental animals have been used to identify key mechanisms governing ocular homeostasis and ocular immune deviation, but the factors responsible for recovery of homeostasis after inflammation are not completely understood (Ohta et al., 1999; Ohta et al., 2000b; Ohta et al., 2000a; J. S. Mo and Streilein, 2001; J.-S. Mo and Wayne Streilein, 2005; O’rourke et al., 1988). One key mechanism that has been identified is the generation of regulatory T cells against ocular and foreign antigens encountered within the eye (Stein-Streilein and Joan, 2008; Taylor, 2007). In the current study, both intraocular antigen load and antigenic memory were found to influence the ability of the local immune microenvironment to recover from inflammation, suggesting they may impact T reg development, trafficking or function in the eye. While the differences were not statistically significant, the concentrations of IL-9 and IL-13 were the only cytokines found in higher concentration on day 1 in unprimed vitreous. IL-9 has traditionally be associated with Th2 response in allergy and helminth infections (Goswami and Kaplan, 2011). But more recently been linked to have been previously associated with immunoregulation of central nervous system autoimmunity in the animal model of multiple sclerosis, experimental autoimmune encephalomyelitis (EAE) (Elyaman et al., 2009; Yoshimura et al., 2020). In EAE, loss of IL-9 led to more severe disease and increased numbers of T cells and activated dendritic cells in the brains of affected animals. Additionally, even though IL-9 synergizes with TGF-β to induce Th17 differentiation, it can also enhance suppressive functions of T reg cells in vivo. Together with these findings in EAE, our data suggests IL-9 expression in the unprimed vitreous may play a protective role in the ocular inflammatory response to Mtb. Future efforts using primed and unprimed animals will help clarify how the baseline context in which the eye experiences an inflammatory event (i.e. novel or known antigen) and the degree of inflammation experienced determines outcome, and identify possible targets that can be modulated to accelerate restoration of immune homeostasis.

While this study focused on the uveitis associated with Mtb infection, chronic post-infectious uveitis has also been described in association with a number of other pathogens (Birnbaum et al., 2012; Zhenyu et al., 2021; Sanghvi et al., 2011; Cunningham et al., 2016) Post-infectious uveitis does not improve with additional antimicrobial therapy but does improve with systemic corticosteroid therapy (Tomkins-Netzer et al., 2018). This response pattern confirms a sterile immune response as opposed to continued viable infection (Filloy et al., 2016; Goldhardt et al., 2016; Wade et al., 2020). Further management strategies for the ensuing chronic uveitis are controversial, and no strategies to prevent chronic post-infectious uveitis are known (Ching Wen Ho et al., 2018). An improved understanding of the mechanisms responsible for chronic post-infectious uveitis in response to Mtb will help identify targets for future therapeutic approaches in this wider range of patients.

In summary, we describe here an expanded characterization of a model of Mtb associated uveitis that demonstrates acute inflammation mediated by innate immune function and chronic uveitis that is characterized by a T cell dominant leukocytic infiltrate and dependent on adaptive immune function. PMU is a new model of chronic post-infectious uveitis that is not dependent on the initiation by immunization with ocular antigens, and may help elucidate new mechanisms of disease in patients with chronic uveitis.

## Supplementary Material

Supplemental Table 2

Supplemental Table 3

Supplemental Table 1

Supplemental Table 4

Supplemental Table 5

Supplemental Figure 1

Supplemental Figure 2

Supplemental Figure 3

Supplemental Figure 4

Supplemental Figure 5

Supplemental Figure 6

Supplemental Figure 7

## Data Availability

Data will be made available on request.
