# Supplementary material for: Systemic prime exacerbates the ocular immune response to heat-killed Mycobacterium tuberculosis
Source: Exp Eye Res. Author manuscript; Available in PMC 2023 Jun 5. (PMC10240933; doi:10.1016/j.exer.2022.109198)
Supplement: Supplemental Table 2 [file NIHMS1899506-supplement-Supplemental_Table_2.pdf]

Supplemental Table 2. Day 7 Vitreous cytokine concentrations					
	Wild type C57BL/6				
	UMU D7		PMU D7		
**Cytokine**	**pg/ml**	**STD**	**pg/ml**	**STD**	**p value**
G-CSF	276.5	279.5	824.4	177.6	**
IL-6	75.5	100.1	119.7	72.6	
IL-17	<2.0	0.0	357.0	115.7	**
Eotaxin	36.3	33.4	138.9	21.3	***
LIF	4.7	4.3	22.1	8.0	**
IP-10	91.6	39.0	1,060.0	67.5	****
KC	18.5	37.7	186.5	48.6	***
MIP-1b	<49.6	0.0	205.9	60.1	**
M-CSF	10.2	4.4	52.4	9.0	***
MIP-1a	56.1	19.8	426.6	95.8	***
MIP-2	<31.5	0.0	230.9	104.5	*
MIG	38.0	27.2	489.2	68.9	****
IL-5	<2.2	0.0	<2.2	0.0	
RANTES	<1.7	0.0	4.9	1.2	**
IL-1b	2.2	0.7	60.9	28.0	**
TNF-a	<2.0	0.0	20.2	6.6	**
1l-12p40	8.5	8.1	63.8	18.7	**
IL-1a	178.7	134.8	359.9	111.9	*
IL-4	<2.3	0.0	<2.3	0.0	
IFN-g	19.3	10.1	42.2	5.6	**
IL-3	<2.1	0.0	3.7	2.4	
MCP-1	8.8	9.1	131.2	34.5	***
IL12-p70	<1.7	0.0	21.1	11.7	*
GM-CSF	4.3	3.4	33.8	12.8	**
IL-15	61.2	17.6	114.9	18.3	**
LIX	36.2	77.1	29.4	51.8	
IL-2	12.9	4.0	17.1	7.1	
IL-7	1.9	0.4	10.9	6.5	*
IL-10	12.1	5.4	9.2	1.7	
VEGF	19.5	6.8	84.1	14.0	***
IL-9	92.4	45.2	228.5	150.2	
IL-13	<3.6	0.0	<3.6	0.0	

