# Supplementary material for: Systemic prime exacerbates the ocular immune response to heat-killed Mycobacterium tuberculosis
Source: Exp Eye Res. Author manuscript; Available in PMC 2023 Jun 5. (PMC10240933; doi:10.1016/j.exer.2022.109198)
Supplement: Supplemental Table 3 [file NIHMS1899506-supplement-Supplemental_Table_3.pdf]

Supplemental table 3. Serum Cytokine concentration

	Serum									
	Naïve		Primed naïve			UMU D7		PMU D7		
Cytokine	pg/ml	STD	pg/ml	STD	p	pg/ml	STD	pg/ml	STD	p
G-CSF	430.5	364.3	2,015	1,279	**	473.2	426.6	1,769	989.8	*
IL-6	2.6	2.1	20.3	32.5		5.2	5.4	9.3	10.6	
IL-17	<19.5	0	<19.5	0		<19.5	0	24.2	8.2	
Eotaxin	946.3	370	772.9	189.9		614.3	300.7	1,142	209.6	***
LIF	<3.6	0	<3.6	0		<3.6	0	<3.6	0	
IP-10	61.75	30.3	114.1	39.1	*	60.5	32.32	145.4	59.5	*
KC	31.32	8.5	55.1	36.2		20.8	16.67	30.9	13.4	
MIP-1b	9.973	8.2	16.3	15.5		10.8	10.75	22.6	13.5	
M-CSF	<2.4	0	<2.4	0		<2.4	0	<2.4	0	
MIP-1a	36.19	43.6	149.6	46.1	****	33.2	38.36	109.7	52.3	*
MIP-2	118.9	28.8	127.7	25.2		122.7	49.02	106.8	55.5	
MIG	147.6	54.3	145.9	59.4		120.5	88.96	189.9	63.7	
IL-5	<22	0	<22	0		<22	0	<22	0	
RANTES	<2.9	0	<2.9	0		<2.9	0	<2.9	0	
IL-1b	<2.5	0	<2.5	0		<2.5	0	<2.5	0	
TNF-a	<3.2	0	<3.2	0		<3.2	0	<3.2	0	
1l-12p40	2.11	0	2.11	0		2.1	0	2.11	0	
IL-1a	287.8	201.4	533.1	259.7		301.5	185	791.9	694.7	
IL-4	<30	0	<30	0		<30	0	<30	0	
IFN-g	<1.6	0	<1.6	0		<1.6	0	<1.6	0	
IL-3	<16	0	<16	0		<16	0	<16	0	
MCP-1	33.4	35.3	41.5	30.1		7.6	5	36.8	20.3	**
IL12-p70	<2.4	0	<2.4	0		<2.4	0	<2.4	0	
GM-CSF	<1.5	0	<1.5	0		<1.5	0	<1.5	1.5	
Il-15	11.46	11.2	15.5	11.2		7.3	8.1	14.2	12	
LIX	-	-	-	-		-	-	-	-	
IL-2	<16	0	<16	0		<16	0	16.8	2.413	
IL-7	<3.6	0	<3.6	0		<3.6	0	<3.6	0	
Il-10	<3.5	0	<3.5	0		<3.5	0	<3.5	0	
VEGF	<2.5	0	<2.5	0		<2.5	0	<2.5	0	
Il-9	33.14	20	98.9	40.2	***	28.1	22.3	48.7	33.1	
Il-13	82.1	33	89.3	22.6		101	43.3	102.3	33.6	
