# Supplementary material for: Systemic prime exacerbates the ocular immune response to heat-killed Mycobacterium tuberculosis
Source: Exp Eye Res. Author manuscript; Available in PMC 2023 Jun 5. (PMC10240933; doi:10.1016/j.exer.2022.109198)
Supplement: Supplemental Table 1 [file NIHMS1899506-supplement-Supplemental_Table_1.pdf]

Supplemental Table 1. Day 1 Vitreous cytokine concentrations

	Wild type C57BL/6										RAG2 deficient	
	Naïve		UMU D1			Primed naïve		PMU D1			PMU D1	
Cytokine	pg/ml	STD	pg/ml	STD	p	pg/ml	STD	pg/ml	STD	p	pg/ml	STD
G-CSF	96.1	87.7	374.3	446.3		255.0	131.7	2,720.0	1,338.0	*	1,089.0	1,104.0
IL-6	3.0	1.6	455.6	543.4		10.5	18.0	3,113.0	1,882.0		1,017.0	925.3
IL-17	<2.01	0.0	4.0	0.7	**	<2.01	0.0	24.6	10.3	*	3.1	0.3
Eotaxin	26.7	17.9	37.5	4.6		31.0	21.7	156.7	50.3	**	41.0	12.9
LIF	<2.79	0.0	129.1	31.7	**	<2.79	0.0	516.0	242.6	*	352.9	373.9
IP-10	22.0	11.4	162.1	81.9	*	22.0	4.4	552.4	204.0	*	143.1	44.2
KC	3.9	2.8	285.7	321.4		6.4	7.5	854.3	615.4		417.0	381.8
MIP-1b	<49.61	0.0	113.6	12.9	***	53.2	6.6	297.4	83.1	**	143.5	59.7
M-CSF	8.0	7.5	126.8	36.4	***	7.9	6.7	299.7	176.4		97.0	45.3
MIP-1a	66.6	23.0	124.1	29.1	*	83.4	51.9	279.6	65.3	**	148.2	87.6
MIP-2	37.3	12.8	2,350.0	1,168.0	*	39.3	12.5	4,979.0	1,837.0	**	3,528.0	2,883.0
MIG	36.8	33.8	51.0	10.0		27.6	19.0	105.2	8.5	***	37.5	3.8
IL-5	<2.16	0.0	5.8	1.5	*	<2.16	0.0	11.6	0.9	****	9.6	1.5
RANTES	<1.8	0.4	4.2	0.3	****	<1.8	0.0	7.3	2.1	**	4.5	0.5
IL-1b	3.9	4.1	169.0	67.3	*	2.6	1.3	282.3	103.0	**	194.8	218.2
TNF-a	<2.0	3.7	22.9	6.1	**	<2.0	0.0	36.6	8.6	**	12.2	3.1
1l-12p40	6.2	4.3	22.5	2.7	***	6.7	5.8	34.7	4.6	****	21.4	1.6
IL-1a	150.8	58.9	462.8	150.4	*	148.7	23.9	674.1	311.3	*	369.0	357.1
IL-4	<2.3	0.0	3.3	0.7		<2.3	0.0	4.6	1.7	****	2.3	0.5
IFN-g	20.5	11.0	33.4	3.9		16.8	3.2	45.3	7.2	***	36.7	10.2
IL-3	<2.1	0.0	4.3	1.3		<2.1	0.0	5.7	0.4	****	2.4	0.2
MCP-1	6.7	6.2	193.4	127.5		5.0	5.5	257.5	122.6	*	250.6	184.0
IL12-p70	2.6	1.8	36.9	1.5	****	2.0	0.4	45.9	4.3	****	38.3	3.4
GM-CSF	5.4	3.0	45.7	1.6	****	6.2	5.0	56.4	5.9	****	48.4	5.4
Il-15	58.8	16.1	158.7	15.3	****	60.3	16.2	185.4	8.4	****	144.6	18.4
LIX	3.9	4.8	220.2	49.6	**	5.0	7.3	254.7	63.6	**	250.5	45.9
IL-2	24.0	11.0	19.7	6.9		15.0	4.9	21.4	2.1		7.6	0.3
IL-7	<1.7	0.0	12.4	3.3	**	2.3	1.2	12.9	0.9	****	18.1	2.3
il-10	18.2	12.1	71.1	27.4	*	17.3	4.9	64.7	8.8	***	51.1	15.3
VEGF	37.9	2.5	31.4	16.7		19.9	10.5	14.7	4.8		4.4	0.7
il-9	51.6	55.3	1,740.0	759.5	*	116.3	110.3	701.7	152.1	***	429.3	28.2
Il-13	7.1	7.7	284.9	112.0	*	<3.6	0.0	99.7	27.7	**	106.1	21.8
