# Supplementary material for: Systemic prime exacerbates the ocular immune response to heat-killed Mycobacterium tuberculosis
Source: Exp Eye Res. Author manuscript; Available in PMC 2023 Jun 5. (PMC10240933; doi:10.1016/j.exer.2022.109198)
Supplement: Supplemental Table 4 [file NIHMS1899506-supplement-Supplemental_Table_4.pdf]

Supplemental Table 4 Criteria for OCT inflammation score		
**Score**	**Anterior Chamber**	**Posterior Chamber**
**NA**	No view beyond anterior cornea	No view of posterior segment
**0**	No inflammation	No inflammation
**0.5**	1–5 cells in the aqueous<br><br>OR corneal edema	Few cells (<50) occupying less than 10% of the vitreous area<br>No subretinal or intraretinal infiltrates or retinal architecture disruption
**1**	6–20 cells in the aqueous<br>OR a single layer of cells on the anterior lens capsule	> 50 cells diffusely (no dense clumps) occupying between 10 and 50% of the vitreous area.<br>No subretinal or intraretinal infiltrates or retinal architecture disruption
**2**	20–100 cells in the aqueous<br><br>OR fewer than 20 cells and a hypopyon present	> 100 cells diffusely (no dense clumps) occupying > 50% of the vitreous area<br>No subretinal or intraretinal infiltrates or retinal architecture disruption
**3**	20–100 cells in the aqueous<br><br>AND a hypopyon OR a pupillary membrane	> 100 cells AND at least one dense vitreous opacity occupying 10–20% of the vitreous area (small opacity)<br>OR > 100 cells and rare (≤ 2) subretinal or intraretinal opacities or retinal pathology
**4**	Any number of aqueous cells<br><br>AND a large hypopyon and pupillary membrane OR anterior structure loss due to severe inflammation	Any amount of individual vitreous cell AND dense vitreous opacity occupying > 20% of the vitreous area (large opacity)<br><br>OR any amount of individual vitreous cell AND many >3 subretinal or intraretinal opacities or other significant retinal pathology
