# Supplementary material for: Systemic prime exacerbates the ocular immune response to heat-killed Mycobacterium tuberculosis
Source: Exp Eye Res. Author manuscript; Available in PMC 2023 Jun 5. (PMC10240933; doi:10.1016/j.exer.2022.109198)
Supplement: Supplemental Table 5 [file NIHMS1899506-supplement-Supplemental_Table_5.pdf]

Supplemental Table 5. Criteria for Histology Score

Criteria	0	1	2
Anterior Chamber (AC) Protein	Scant acellular particles staining with eosin in the AC	Moderate, but not confluent, extracellular eosin staining anywhere in the AC	Confluent or near confluent extracellular eosin staining throughout the AC
Anterior Chamber (AC) Cell	No cells	1–100 cells, but no dense aggregations of cells	>100 cells, or dense aggregations of cells
Ciliary Body Inflammation	No leukocyte infiltration of the ciliary body or surrounding vitreous	Unilateral presence of leukocytes infiltrating the ciliary body and/or the surrounding vitreous.	Bilateral presence of leukocytes infiltrating the ciliary body and/or the surrounding vitreous.
Vitreous celll	rare to no cells	1–100 cells, but no dense aggregations of cells	> 100 cells or dense aggregations of cell
Retinal Vascular Inflammation	No retinal vessels with perivascular leukocytes	One vessel per section with perivascular leukocytes	>1 vessel per section with perivascular leukocytes
Retinal fold or damage	No retinal damage	1–3 retinal folds per section	>3 retinal folds per section, or any other retinal layer destruction or intraretinal hemorrhage
