# Supplementary figures and images for: Systemic prime exacerbates the ocular immune response to heat-killed Mycobacterium tuberculosis
Source: Exp Eye Res. Author manuscript; Available in PMC 2023 Jun 5. (PMC10240933; doi:10.1016/j.exer.2022.109198)

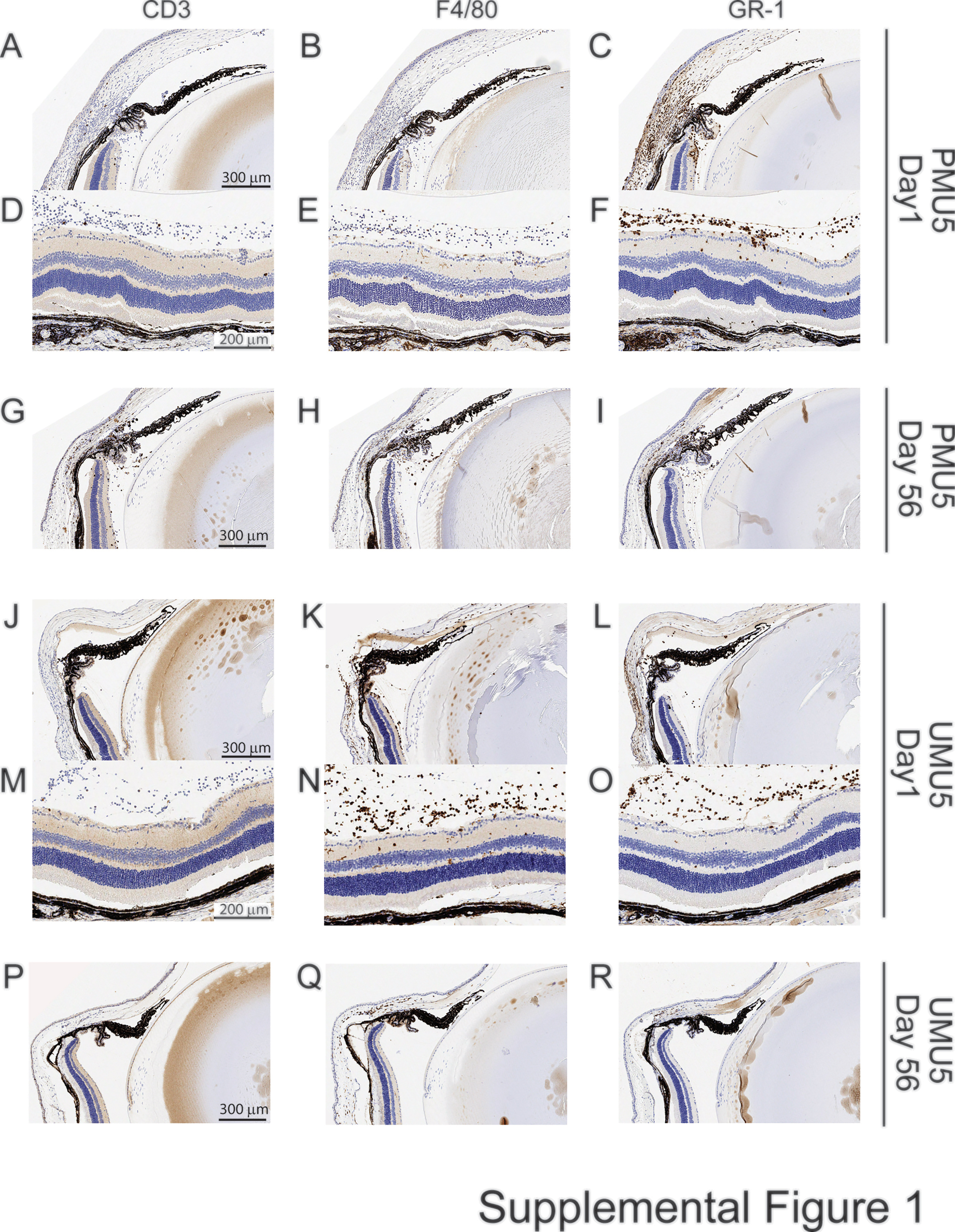

Supplement: Supplemental Figure 1 [file NIHMS1899506-supplement-Supplemental_Figure_1.jpg]

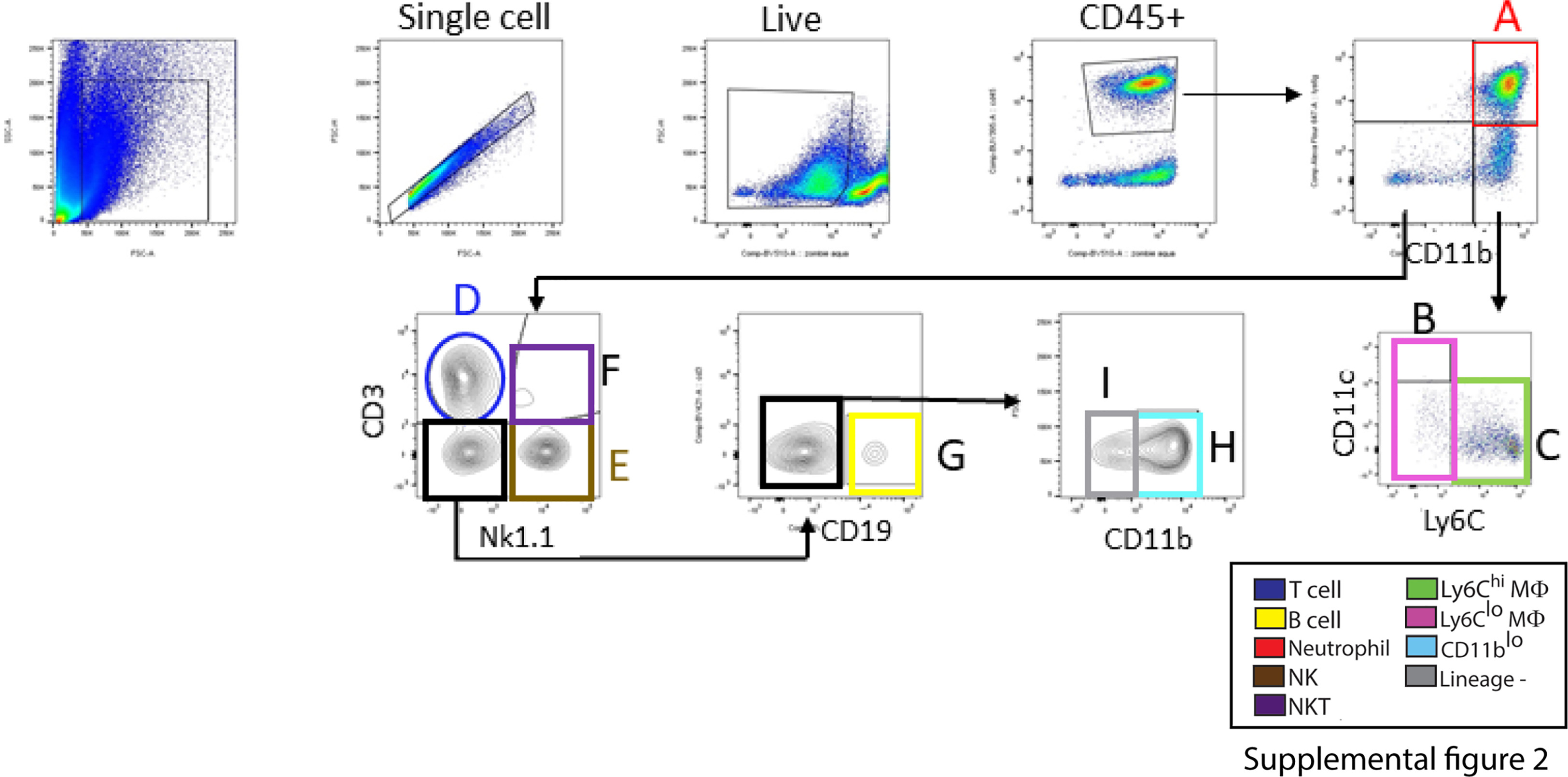

Supplement: Supplemental Figure 2 [file NIHMS1899506-supplement-Supplemental_Figure_2.jpg]

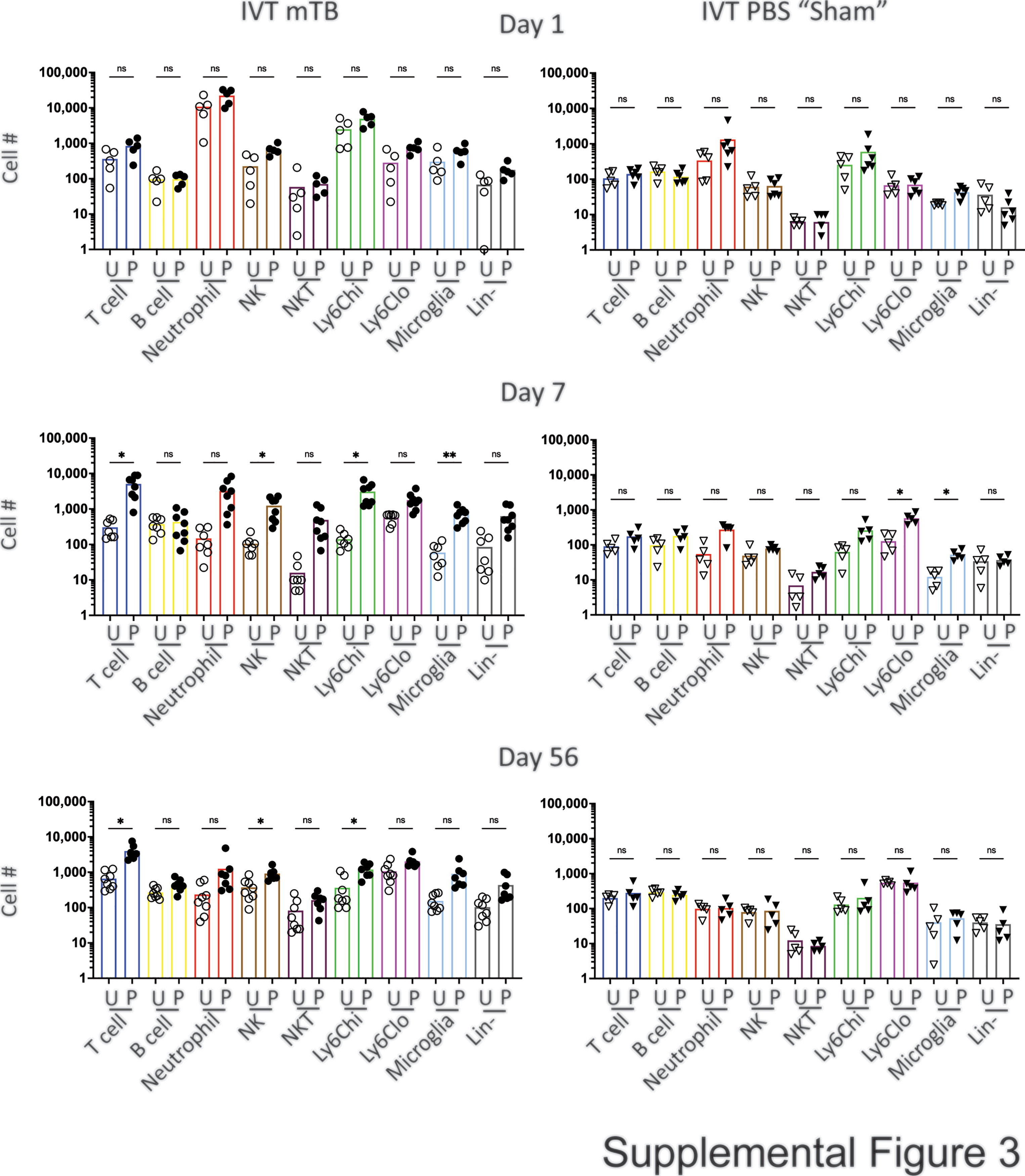

Supplement: Supplemental Figure 3 [file NIHMS1899506-supplement-Supplemental_Figure_3.jpg]

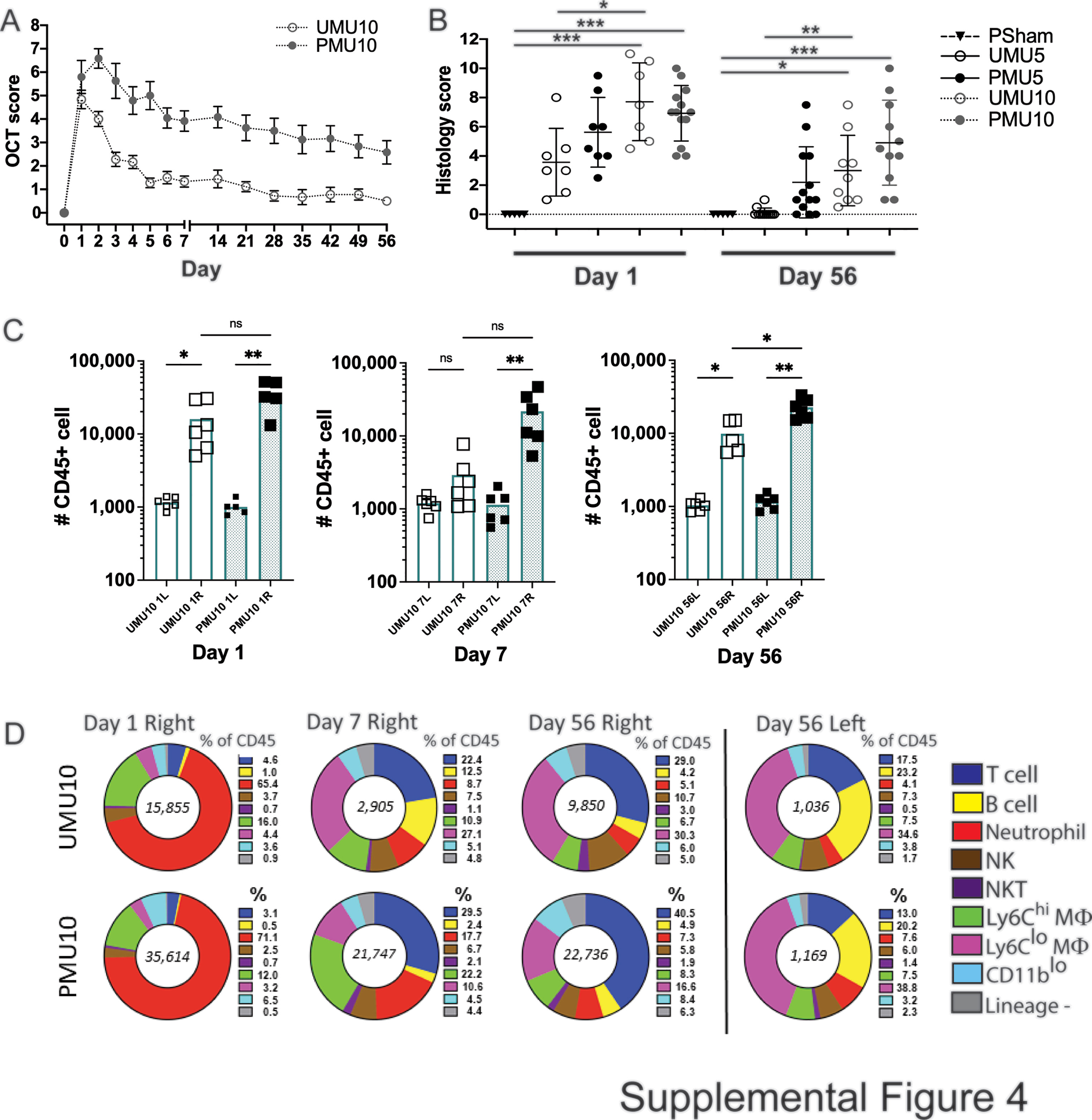

Supplement: Supplemental Figure 4 [file NIHMS1899506-supplement-Supplemental_Figure_4.jpg]

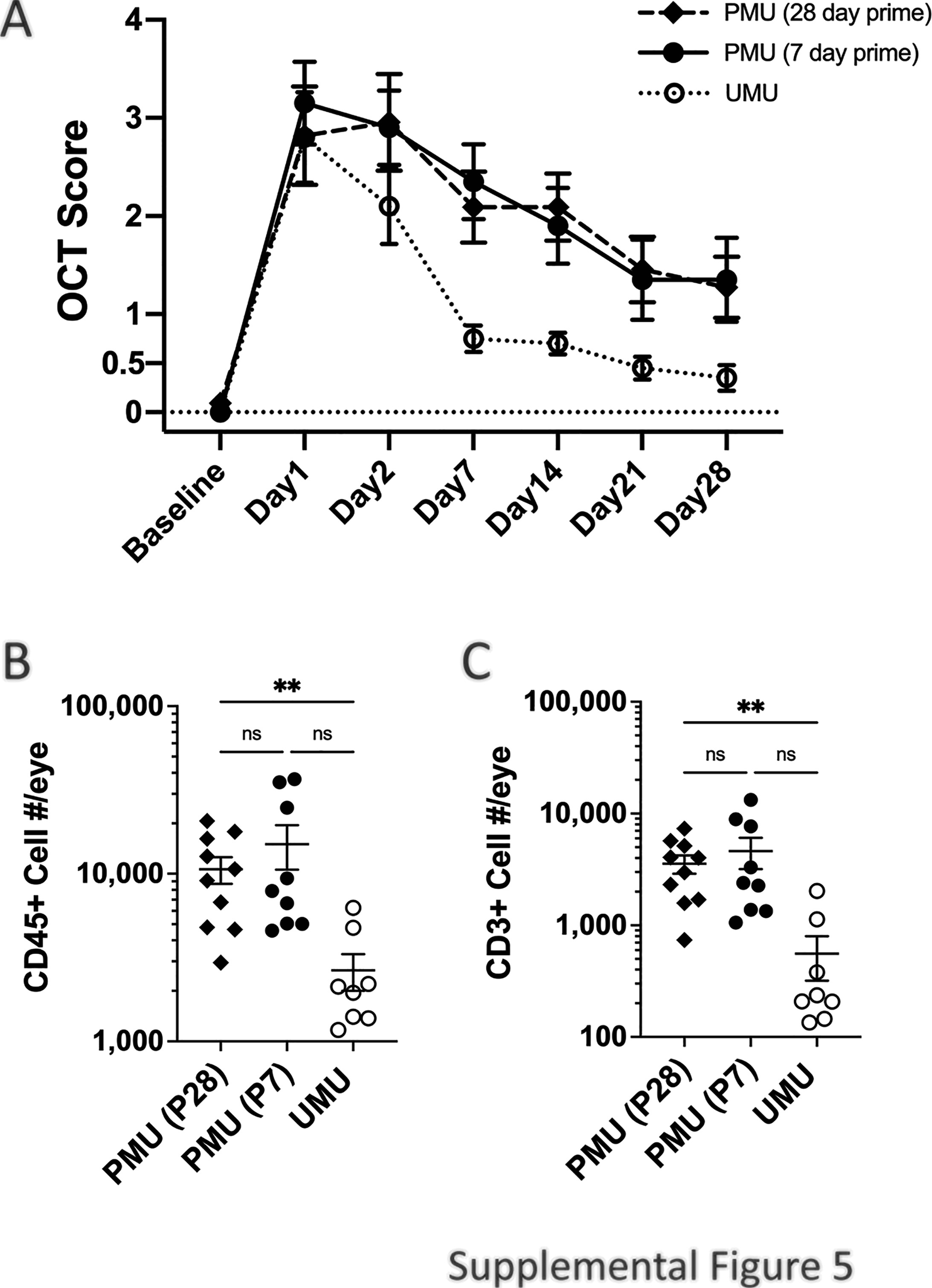

Supplement: Supplemental Figure 5 [file NIHMS1899506-supplement-Supplemental_Figure_5.jpg]

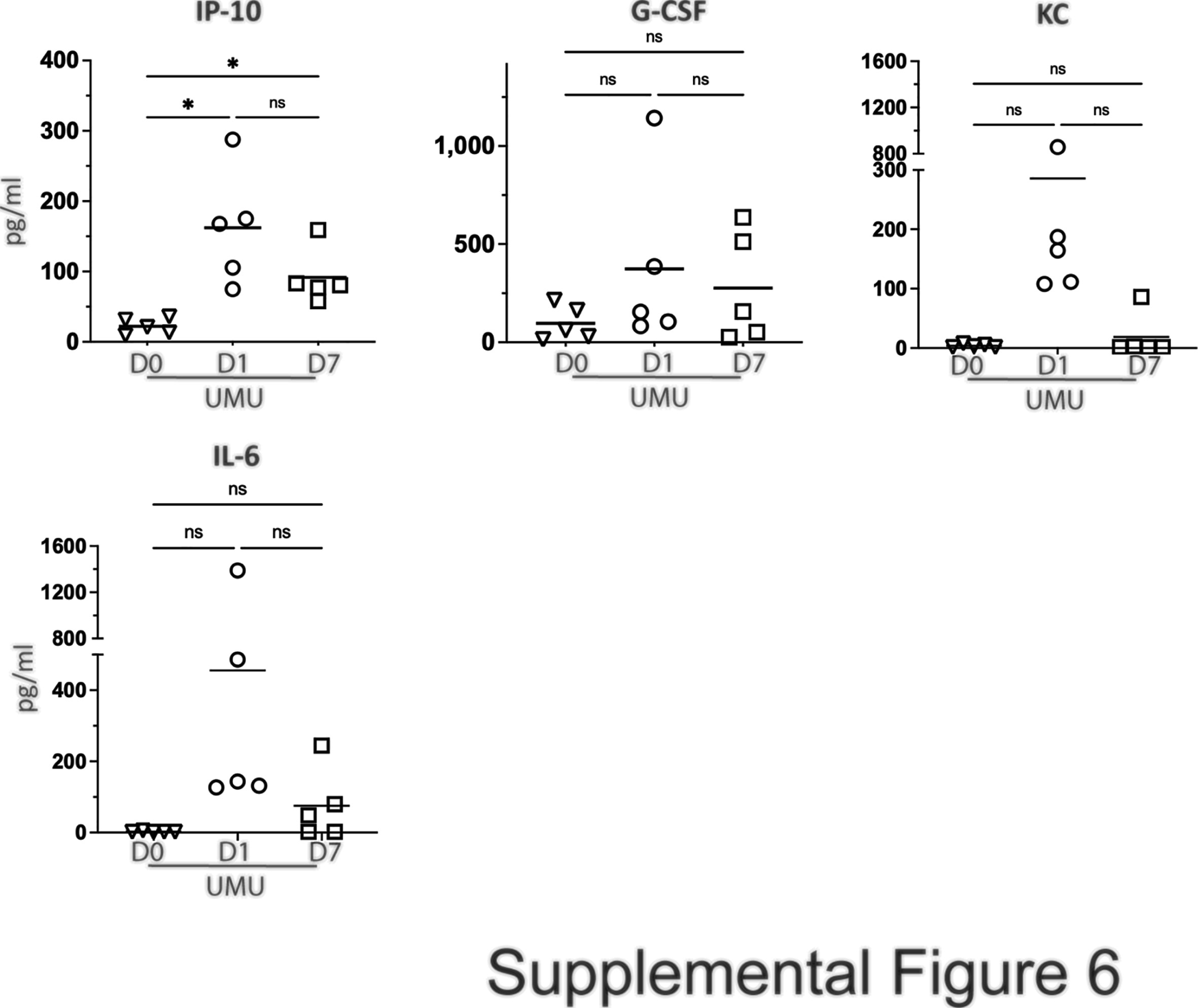

Supplement: Supplemental Figure 6 [file NIHMS1899506-supplement-Supplemental_Figure_6.jpg]

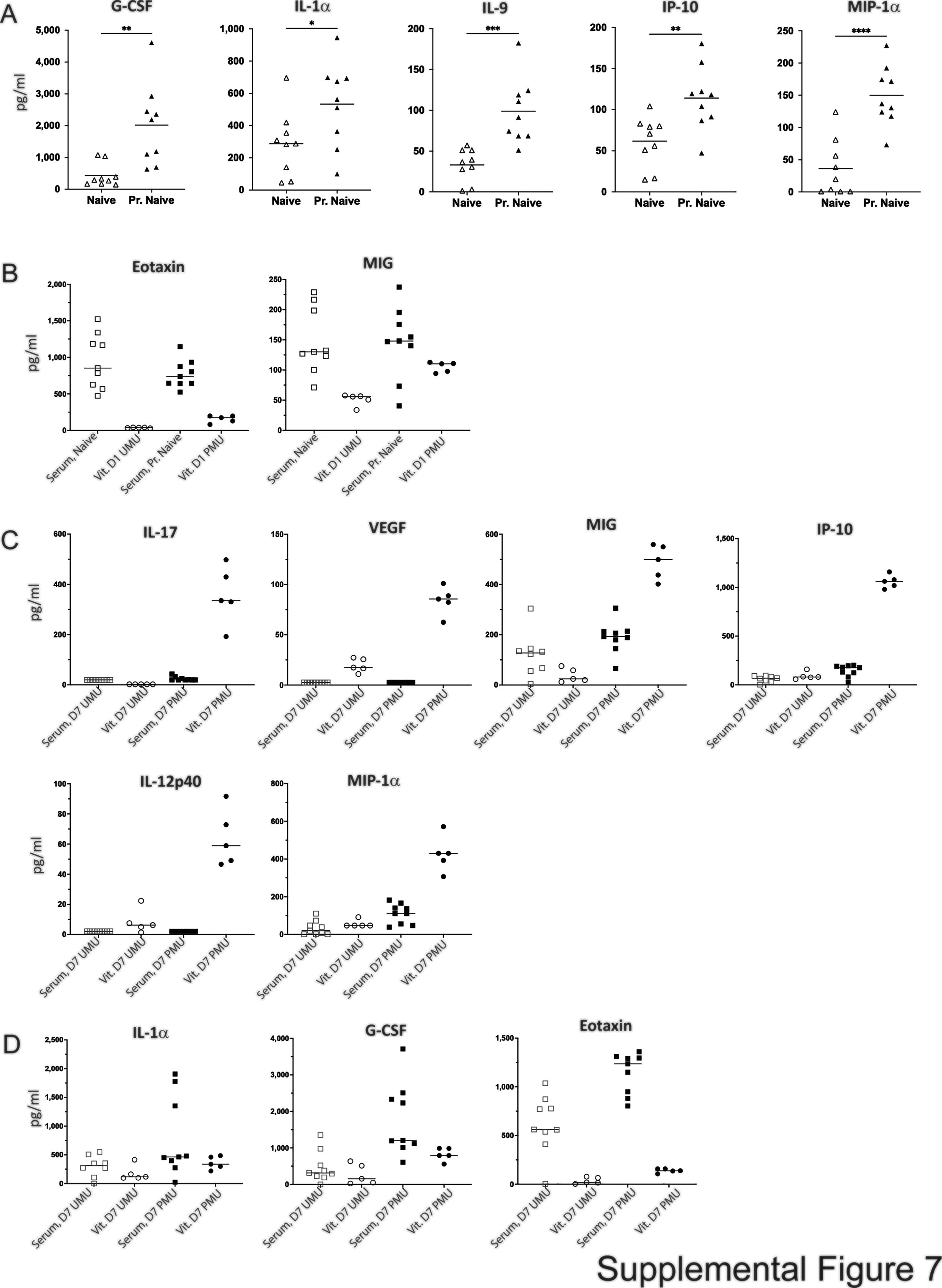

Supplement: Supplemental Figure 7 [file NIHMS1899506-supplement-Supplemental_Figure_7.jpg]
